# Analysis of the Circadian Regulation of Cancer Hallmarks by a Cross-Platform Study of Colorectal Cancer Time-Series Data Reveals an Association with Genes Involved in Huntington’s Disease

**DOI:** 10.3390/cancers12040963

**Published:** 2020-04-13

**Authors:** Müge Yalçin, Rukeia El-Athman, Koliane Ouk, Josef Priller, Angela Relógio

**Affiliations:** 1 Institute for Theoretical Biology (ITB), Charité—Universitätsmedizin Berlin, corporate member of Freie Universität Berlin, Humboldt-Universität zu Berlin, and Berlin Institute of Health, 10117 Berlin, Germany; muege.yalcin@charite.de (M.Y.); rukeia.el-athman@bihealth.de (R.E.-A.); 2 Molecular Cancer Research Center (MKFZ), Medical Department of Hematology, Oncology, and Tumour Immunology, Charité—Universitätsmedizin Berlin, corporate member of Freie Universität Berlin, Humboldt-Universität zu Berlin, and Berlin Institute of Health, 10117 Berlin, Germany; 3 Department of Neuropsychiatry and Laboratory of Molecular Psychiatry, Charité-Universitätsmedizin Berlin, 10117 Berlin, Germany; koliane.ouk@charite.de (K.O.); josef.priller@charite.de (J.P.); 4 German Center for Neurodegenerative Diseases (DZNE), 10117 Berlin, Germany; 5 Dementia Research Institute (DRI), University of Edinburgh and UK DRI, Edinburgh EH16 4SB, UK; 6 Department of Human Medicine, Institute of Systems Medicine and Bioinformatics, MSH Medical School Hamburg—University of Applied Sciences and Medical University, 20457 Hamburg, Germany

**Keywords:** circadian clock, colorectal cancer, drug targets, high-throughput time course data, Huntington’s disease

## Abstract

Accumulating evidence points to a link between circadian clock dysfunction and the molecular events that drive tumorigenesis. Here, we investigated the connection between the circadian clock and the hallmarks of cancer in an in vitro model of colorectal cancer (CRC). We used a cross-platform data normalization method to concatenate and compare available microarray and RNA-sequencing time series data of CRC cell lines derived from the same patient at different disease stages. Our data analysis suggests differential regulation of molecular pathways between the CRC cells and identifies several of the circadian and likely clock-controlled genes (CCGs) as cancer hallmarks and circadian drug targets. Notably, we found links of the CCGs to Huntington’s disease (HD) in the metastasis-derived cells. We then investigated the impact of perturbations of our candidate genes in a cohort of 439 patients with colon adenocarcinoma retrieved from the Cancer Genome Atlas (TCGA). The analysis revealed a correlation of the differential expression levels of the candidate genes with the survival of patients. Thus, our study provides a bioinformatics workflow that allows for a comprehensive analysis of circadian properties at different stages of colorectal cancer, and identifies a new association between cancer and HD.

## 1. Introduction

Multiple biological processes that drive the onset and progression of malignant growth have been extensively investigated and termed as hallmarks of cancer [1]. These hallmarks represent key biological events that are rewired by tumors and favor survival of malignant cells in the host tissue. Accumulating evidence highlights the need of targeting these molecular alterations in cancer treatment [2,3]. Although available chemotherapeutic treatment strategies have been partially successful in eliminating cancer cells, the resulting severe side effects (i.e., fatigue, hypertension and liver toxicity), increased therapy resistance and relapse of disease reveal the diversity of therapeutic outcome due to inter- and intra-patient variability [4]. These findings have directed the cancer research field towards the development of patient-specific treatment strategies [5].

A promising link has been reported between the circadian clock and the key molecular events that contribute to tumorigenesis [6,7], which is likely to provide a promising temporal component to personalized therapy [8]. The mammalian circadian clock describes an internal, self-sustained timekeeping mechanism that regulates many aspects of physiology and behavior [9], and enables organisms to anticipate and respond to environmental changes such as light-dark cycles [10]. This ensures the appropriate timing of biological processes including the regulation of core body temperature, sleep/wake cycles, immunological activity and metabolism [11,12].

At the cellular level, the circadian clock is driven by transcriptional and translation feedback loops (TTFLs) [13,14], comprising gene and protein families of transcriptional repressors (*PER*, *CRY*, *REV-ERB*) and activators (*CLOCK*, *BMAL*, *ROR*) [15,16]. These elements drive 24 h rhythmic oscillations in the expression of target genes, the so-called clock-controlled genes (CCGs). Oscillations of core-clock genes and CCGs affect various cellular mechanisms, including the cell cycle, DNA damage response, and cell death, which contribute to tumor initiation and progression [17,18,19]. In a healthy cellular state, the cell cycle is regulated strictly to protect genomic fidelity during DNA synthesis and cell division [20]. Circadian regulation of the cell cycle was reported to occur via the modulation of cell cycle checkpoints such as the *Wee1* kinase [21] that regulates G2-M transition, *Myc* [22,23], *p53* [24,25], *p21* [26], and *p16* [27,28]. Disruption of core-clock components was found to be associated with accelerated tumor growth in several mouse models including malignant lymphoma [29], lung [30], colorectal cancer [26], ovarian, pancreatic, and intestinal cancer [18].

The investigation of, potentially dysregulated, clock phenotypes in cancer requires the analysis of circadian rhythmicity at the transcriptome level. Although the availability of high-throughput cancer data sets has increased in the last years, most of this data was obtained at a single time point rather and not sampled over a circadian day and is thus inadequate for circadian analyses.

In the current study, we illustrate the link between the circadian clock and the hallmarks of cancer in a meta-analysis of an in vitro model of colorectal cancer (CRC). For our analysis, we used available microarray and RNA-sequencing (RNA-seq) time series data of two cell lines that are derived from a primary tumor (SW480) and a lymph node metastasis (SW620) of the same patient. We developed a data analysis workflow for the cross-platform comparison and concatenation of the time series datasets. This yielded a longer time-series and allowed for more robust results concerning circadian gene sets, related circadian parameters and the subsequent analysis regarding significantly phase-clustered biological pathways and relevant clock-regulated genes in the tumorigenesis process.

In the concatenated data set, we identified robust sets of 24 h rhythmic genes. A phase set enrichment analysis (PSEA) revealed phase-clustered biological pathways that differ between the primary tumor and the metastasis-derived cells. In SW480 cells, enriched biological pathways included DNA repair mechanisms, proliferative pathways such as MAPK, WNT, and JAK-STAT and immunological response such as antigen presentation. In the metastasis-derived SW620 cells, biological pathways that are known to play a role in transcriptional regulation (i.e., RNA polymerase, basal transcription factors and ubiquitin-mediated proteolysis) were enriched for circadian genes with similar phases. Surprisingly, we also found phase-clustered pathways related to Huntington’s disease (HD) in the metastatic cells. We extended our rhythmicity analysis to identify circadian drug target genes [31] and found 19 oscillating drug targets in total. In particular, we showed that *MTOR* and *AKT1* are oscillating in our data sets and associated to the circadian clock, cancer hallmarks and circadian drug targets.

We studied the impact of candidate genes from the merged lists for the extended core clock network (ECCN) [32], HD, cancer hallmarks and circadian drug targets in an independent colon adenocarcinoma clinical study that was obtained from TCGA. We plotted a graphical summary of mutational frequencies in colon adenocarcinoma patients (439 samples). Although we could not observe a significant impact on patient survival, our results showed that 4 of the top frequently mutated candidate genes were also involved in HD. These candidate genes were *HSPH1*, *HTT*, *SIN3A*, and *CLTC*. Alterations in expression of our candidate genes showed correlation with patient survival in core clock components *PER3*, *NR1D1*, and *CSNK2A1* and angiogenesis related *VEGFA*. Furthermore, our analysis highlights the link between the core-clock genes to cancer hallmark genes such as *AKT1*, *MTOR*, and *MYC* based on the literature. Further investigation of these genes might be helpful to establish alternative treatment regimens for cancer patients by considering the circadian clock.

## 2. Results

### 2.1. Correlation of Gene Expression between Circadian Microarray and RNA-seq Data of Human CRC Cell Lines

We evaluated the circadian transcriptome in an in vitro CRC progression model of the CRC cell lines SW480 and SW620, previously profiled by time-series DNA microarrays [33] and RNA-seq of mRNAs [34], and aimed to concatenate the datasets to gain a longer and more robust circadian time series in order to explore putative cancer-relevant circadian pathways. The microarray dataset consists of nine samples that were taken from 0 to 24 h after synchronization of the cells by medium change, whereas the eleven RNA-seq samples were taken from 12 to 42 h after synchronization. Both the samples from the microarray, as well as from the RNA-seq datasets were previously produced by our group. Different methods can be used to synchronize the cell population before circadian measurements (e.g., serum shock, use of dexamethasone), and we previously tested different synchronization methods for these cells [27,35,36,37]. As a simple medium change led to comparable results in our cell lines, we decided to use the simplest and less interfering way for cell synchronization [33,34,38]. For the generation of the data sets used in the work, cells were synchronized by medium change.

The microarray dataset covers a full circadian cycle with one additional time replicate of the first time point (0 h = 24 h) and the RNA-seq datasets covers 1.25 circadian cycles with time replicates of three time points (12 h = 36 h; 15 h = 39 h; 18 h = 42 h).

**Figure 1:**
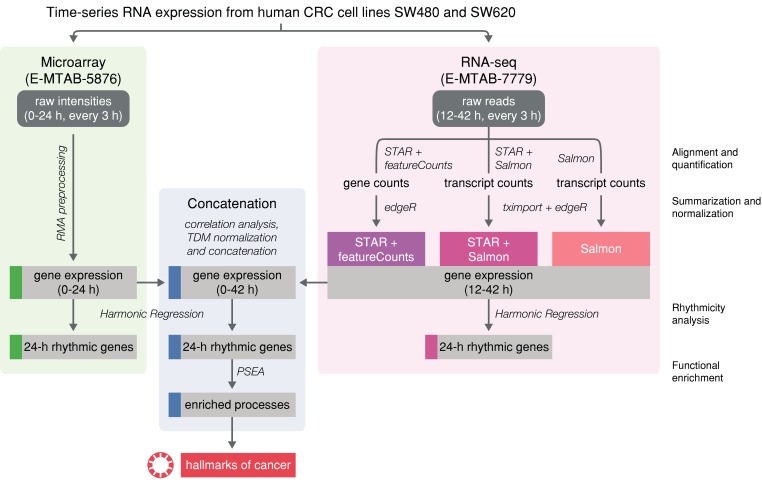
Workflow for the comparison of time-series microarray and RNA-seq data from human colorectal cancer (CRC) cell lines. Two circadian datasets (microarray and RNA-seq) for the cell lines SW480 and SW620 were pre-processed to determine gene expression values. For the RNA-seq data, three different methods (*STAR + featureCounts*, *STAR + Salmon*, *Salmon*) for the alignment and quantification of reads were applied to determine gene expression values. A correlation analysis for shared time points revealed which RNA-seq pre-processing method yields expression values that correlate best with the microarray expression. The best-correlated RNA-seq expression dataset was then TDM-transformed to the range of the microarray data and both datasets were concatenated to gain a longer time-series. 24 h rhythmic gene sets were determined based on the three different sets of data (microarray, RNA-seq and concatenated). Biological pathways enriched for the 24 h rhythmic genes of the concatenated dataset were determined and investigated with view to hallmarks of cancer.

Both datasets were re-analyzed and compared to investigate how well 24-h rhythmic genes, associated circadian parameters, and circadian pathways are replicated across platforms (Figure 1). While for microarray data, the Robust Multi-array Average (RMA) method is commonly used to gain expression values, no standard method for the pre-processing of circadian RNA-seq data has so far been established [39]. For this reason, a tripartite pipeline was used for the alignment and quantification of the raw RNA-seq reads: Two of the chosen pipelines employ the STAR aligner for the alignment of the raw reads to the human genome. Subsequently, the aligned reads were either quantified by featureCounts (*STAR + featureCounts*) or by Salmon in alignment-based mode (STAR + Salmon). In the third pipeline, reads were directly quantified by Salmon in quasi-mapping-based mode (Salmon). The average mapping rates of STAR (81.7% ± 2.55) and Salmon (87.1% ± 0.95) are comparable and consistent across time points and cell lines (Figure S1A). An expression cut-off of at least 0.5 CPM per gene on average over all time points resulted in 13,904 to 16,478 expressed genes in SW480 cells and 13,495 to 15,933 expressed genes in SW620 cells, depending on the pre-processing method (Figure S1B). For both cell lines, *STAR + featureCounts* yields the highest number of expressed genes, followed by *STAR + Salmon*. Independent of the chosen method, the number of expressed genes in SW480 cells is higher than the respective number in SW620 cells. Analogously, different cut-offs for expressed genes were compared for the microarray data, resulting in 21,798 expressed genes with a minimum mean log_2_ RMA-pre-processed intensity value of 3 across all time points in SW480 cells and 21,764 genes in SW620 cells (Figure S1C). Since there are no universal cut-offs to exclude genes with low expression values either for microarray intensity values or RNA-seq counts, the chosen cut-offs are arbitrary and could be adjusted to gain expressed gene sets of comparable sizes for the different platforms. For the present study, this is not necessary because we focus on genes that are commonly expressed across platforms in subsequent correlation and rhythmicity analyses.

**Figure 2:**
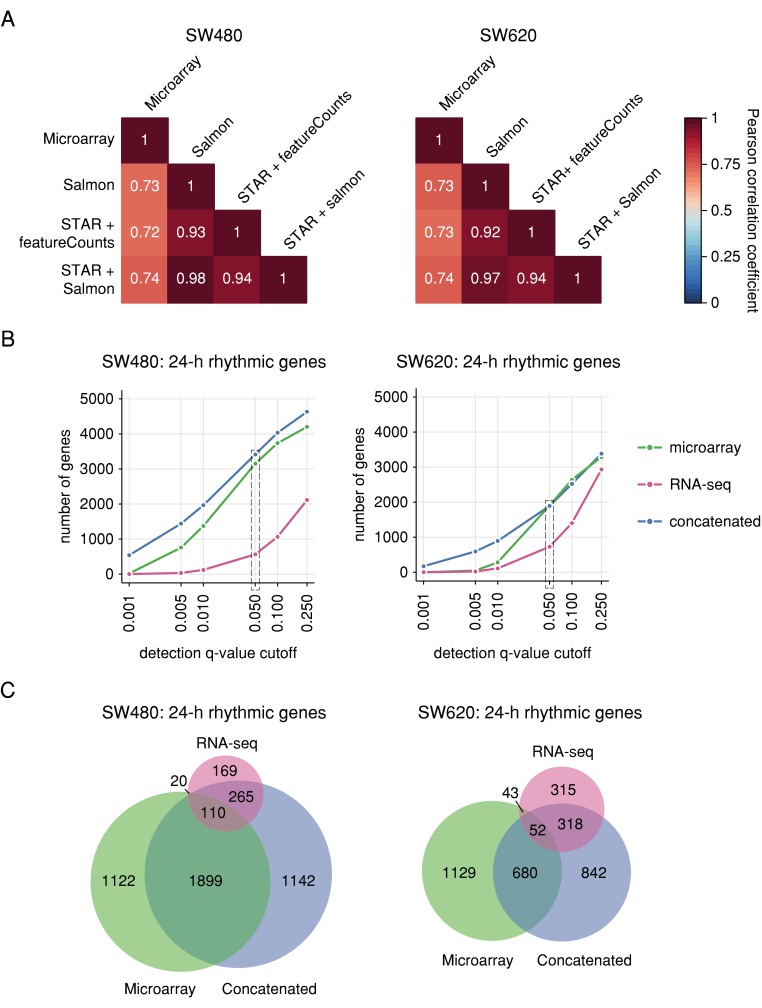
Correlation of gene expression and intersection of 24 h rhythmic gene sets between circadian microarray and RNA-seq data of human CRC cell lines. (**A**) Mean Pearson correlation coefficients between expression values determined by different platforms and different downstream methods for SW480 cells (left panel) and SW620 cells (right panel). Expression values were determined based on microarray data and RNA-seq data (*Salmon*, *STAR + featureCounts*, *STAR + Salmon*). Shown are the mean Pearson correlation coefficients between gene expression values determined for identical time points (12–24 h since synchronization). (**B**) Number of 24 h rhythmic genes for SW480 cells (left panel) and SW620 cells (right panel) based on the three datasets (microarray: green; RNA-seq: pink; concatenated: blue) as determined by harmonic regression for different q-value cut-off and a relative amplitude cut-off of 0.1. The grey rectangle marks the cut-off chosen to determine 24 h rhythmic gene for subsequent analyses. (**C**) Intersections between 24 h rhythmic gene sets in SW480 cells (left panel) and SW620 cells (right panel) identified based on microarray data (green), RNA-seq data (pink) and the concatenated expression of both methods (blue).

For all commonly expressed genes and shared sampling time points, we compared the resulting RNA-seq expression values to each other and to the microarray expression values in a correlation analysis (Figure 2A). Expression values determined by the three RNA-seq analysis pipelines have high mean Pearson correlation coefficients among each other (> 0.9) and are similar across cell lines. We observed the highest correlation between STAR + Salmon and Salmon for both cell lines, indicating that the alignment/quasi-mapping step has a smaller impact on resulting gene expression values than the subsequent quantification of the mapped reads. The mean correlation coefficients between the three RNA-seq pipelines and the microarray data are very similar across methods and cell lines as well, but lower (ranging from 0.72 to 0.74). This result indicates that the choice of the RNA-seq analysis pipeline does not have a large influence on the correlation coefficients, which are primarily determined by the chosen platform. Nonetheless, since the highest correlation of the three pipelines with the microarray data is observed for the *STAR + Salmon* analysis for both cell lines, we consider the *STAR + Salmon* dataset to represent the best approximation to the microarray data for subsequent analyses and hereafter denote it as “RNA-seq data”.

### 2.2. Cross-Platform Concatenation of Time Series Reveals Robustly Circadian Gene Sets for SW480 and SW620 Cells

Since the microarray and RNA-seq datasets contain overlapping but not identical time series, a concatenation of the data would yield a longer time series of nearly two circadian cycles (42 h) with a consistent sampling resolution of 3 h. However, independent of the correlation of gene expression, the range of the abundance measure of the two methods is different: while microarrays measure fluorescence intensities and yield RMA-pre-processed expression values in a log_2_-scaled range from 0 to 13.5, RNA-seq quantifies gene expression in CPM in a higher dynamic log_2_-range from −8 to 15 (Figure S2A). To circumvent this dataset shift and to make expression values comparable across platforms, we applied TDM-normalization to the RNA-seq data [40]. The TDM method transforms an RNA-seq dataset to have a similar distribution as a microarray dataset while keeping inter-observation dependencies intact. Indeed, the distribution of the TDM-transformed RNA-seq expression data closely resembles that of the microarray data for both cell lines (Figure S2A). However, when comparing mean expression levels over the complete time series of individual genes, we still observe a tendency for a lower mean expression of lowly expressed genes and a higher mean expression of highly expressed genes for the RNA-seq data which is likely due to its higher dynamic range (Figure S2B). Nonetheless, for the bulk of genes, the gene expression values of both platforms are comparable after the TDM-transformation of the RNA-seq data, thus allowing for a concatenation of the datasets.

We applied the harmonic regression method to identify 24 h rhythmic gene sets for all three datasets, using an FDR of 5% and a relative amplitude cut-off of 0.1 (Figure 2B). When comparing the overlaps between the 24-h rhythmic gene sets identified based on the two different platforms and the concatenation of both, it becomes apparent that both the number and the identity of rhythmic genes differ greatly between the methods (Figure 2C). The intersection of all three datasets only amounts to 110 (2.3%) genes commonly identified as 24 h rhythmic in SW480 cells and 52 (1.5%) in SW620 cells. For both cell lines, few genes were commonly identified as rhythmic by the microarray and RNA-seq data but not by the concatenated data, indicating that the concatenation of the two-time series successfully captures the circadian transcriptome identified by either one of the platforms. Additionally, many genes were identified as significantly 24 h rhythmic in the concatenated data that were not detected in either one of the two shorter time series, possibly capturing false negative circadian genes. Contrary to expectations and previous results gained from the microarray data [33], more 24 h rhythmic genes were identified for SW620 cells (728) than for SW480 cells (564) based on the RNA-seq data. In contrast, we detected a higher number of 24 h rhythmic genes in SW480 (3426) than in SW620 cells (1892) based on the concatenated dataset.

In the next step, we compared circadian parameters in a gene-wise manner for all genes that were identified to be 24 h rhythmic in the same cell line in at least two of the datasets. The phases estimated based on microarray and RNA-seq data of SW620 cells have a lower absolute circular Pearson correlation coefficient (0.05) than those estimated for SW480 cells (0.26) and are not significantly correlated (*p* = 0.58) (Figure S3A). The correlation between the phases estimated based on the concatenated data and the microarray data is much higher (circular Pearson correlation coefficient 0.99 for SW480 cells and 0.85 for SW620 cells) and significant (*p* < 0.05) (Figure S3B), as is the correlation between the concatenated phases and the RNA-seq phases (circular Pearson correlation coefficient 0.95 for SW480 cells and 0.87 for SW620 cells) (Figure S3C). The lowest gene-wise correlation of relative amplitude values was observed between amplitudes estimated based on the RNA-seq and the microarray data for both cell lines (Pearson correlation coefficient 0.28 for SW480 cells and 0.20 for SW620 cells) (Figure S3D). The amplitude correlation is highest between the microarray and the concatenated data (Pearson correlation coefficient 0.83 for SW480 cells and 0.77 for SW620 cells) (Figure S3E) and considerably lower between RNA-seq data and concatenated data (Pearson correlation coefficient 0.32 for SW480 cells and 0.48 for SW620 cells), where a trend for higher amplitudes can be observed for the RNA-seq data (Figure S3F). Overall, the cross-platform concatenation of time series yielded 24-h rhythmic gene sets with more robust circadian parameters than simply comparing circadian gene sets separately derived from microarray and RNA-seq data.

**Figure 3:**
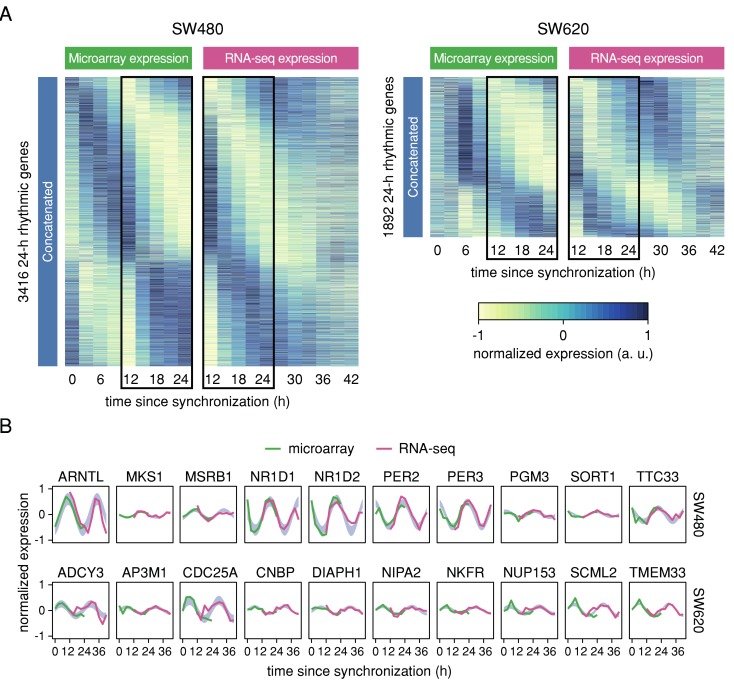
Concatenation of samples from circadian microarray and RNA-seq datasets reveals robustly 24-h rhythmic genes across platforms. (**A**) Range-normalized, phase-ordered microarray (respective left panel) and RNA-seq expression (respective right panel) heatmaps of genes that were identified as 24-h rhythmic in SW480 cells (two leftmost panels) and SW620 cells (two rightmost panels) in the concatenated data. Each row represents one gene. Phases were estimated based on the concatenated data. Black rectangles mark the shared time points between the microarray and the RNA-seq data. (**B**) Normalized time-series expression of the top ten 24 h rhythmic genes identified in SW480 cells (top row) and SW620 cells (bottom row) based on the concatenated data. Microarray expression values are represented by green lines and RNA-seq expression values by pink lines. The blue area marks the confidence area of the harmonic regression fitted to the concatenated data for 24-h rhythmic genes.

We visualized the expression of the complete set of 24 h rhythmic genes identified based on the concatenated data across platforms by phase-ordered heatmaps (Figure 3A). Gene expression of the overlapping time points is very similar between microarray and RNA-seq data, indicating that the cycling genes are indeed reproducible across platforms. However, for the RNA-seq data, the second peak/trough of expression is less distinct than the first for both cell lines. This might be due to the fact that the heatmaps are ordered according to the phase estimated based on the concatenated data set and not based on the individual platforms. Alternatively, it could also result from a progressive loss of synchrony between the cells due to the lack of a synchronizing stimulus. Five out of the top ten 24 h rhythmic genes identified for SW480 cells based on the concatenated data are core clock genes: *BMAL1*, *NR1D1*, *NR1D2*, *PER2*, and *PER3* (Figure 3B). All display very similar oscillations between the two platforms, indicating that the normalization and concatenation of the data is suitable for the detection of robustly circadian genes. No core clock genes are detected among the top ten 24-h rhythmic candidate genes detected in SW620 cells (Figure 3B), but one of the candidate genes, *CDC25A*, is a target of the core clock protein *NPAS2* and known to promote cell survival of hepatocellular carcinoma [41]. The candidate circadian gene *NUP153* which is encoding for a nuclear pore protein has been found to be involved in the control of the nuclear translocation of the *Drosophila* clock proteins PER and TIM [42].

### 2.3. Oscillatory Expression of Cancer Hallmark-Associated Genes and Drug Target Genes Differs between the CRC Cell Lines

**Figure 4:**
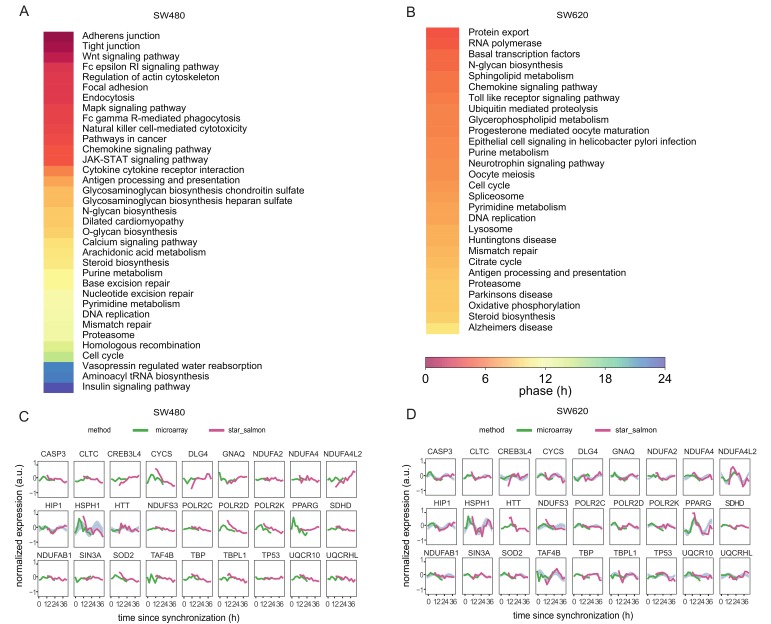
Phase-clustered circadian pathways in SW480 and SW620 cells. Circadian-phase-clustered KEGG pathways enriched for 24 h rhythmic gene sets identified for SW480 cells (**A**) and SW620 cells (**B**). The colors represent the vector-average phases of the pathways. Normalized time series expression of the HD associated genes (27 genes) in SW480 cells (**C**) and SW620 cells (**D**) based on the concatenated data. Microarray expression values are represented by green lines and RNA-seq expression values by pink lines. The blue area marks the confidence area of the harmonic regression fitted to the concatenated data for 24 h rhythmic genes.

We used the PSEA to identify significantly phase-clustered biological pathways enriched for the 24 h rhythmic genes in SW480 and SW620 cells (*q* < 0.05 determined by Kuiper test against a uniform distribution). While for SW480 cells, enriched pathways had mean phases distributed across the whole circadian cycle from 0 to 23.6 h, phases were clustered between 4.5 and 10.3 h, for SW620 cells. For both CRC cell lines, the enriched pathways included cancer-relevant processes, such as cell cycle, DNA repair, and DNA replication (Figure 4A). Several of the pathways have previously been identified to be phase-clustered based on the microarray data, though partly with different phases [33]. Other biological pathways enriched in SW480 cells were involved in extracellular matrix organization and intercellular communication, immune response, growth pathways such as MAPK and DNA damage response (Figure 4A). In SW620 cells, the analysis showed enriched biological processes involved in protein biosynthesis and the structure of plasma membrane (Figure 4B). Interestingly, several neurodegenerative diseases and neuronal communication pathways were also enriched in SW620 cells. These included Alzheimer’s disease (AD), Parkinson’s disease (PD), and Huntington’s disease (HD). These disorders are characterized by neurodegeneration and commonly show dysregulation of circadian rhythms of physiological and behavioral processes, such as hormone secretion, blood pressure, body temperature, activity, and sleep-wake cycle [43]. Notably, HD has been associated with lower cancer risk [44,45]. HD is caused by a CAG trinucleotide repeat expansion in the huntingtin (*HTT*) gene, resulting in a protein with an expanded polyglutamine tract. Although HD is caused by the mutation of one single gene, multiple other genes have been reported to contribute to the HD pathogenesis. Pathways usually described as linked to HD include cytotoxicity, apoptosis, and calcium signaling, but processes and pathways associated with cancer such as cell cycle, RNA splicing, Wnt, and erbB signaling are also significantly enriched in HD patients and mouse models of the disease [46]. For this reason, we next explored whether HD-related genes were oscillating in our dataset (Figure 4C,D). We extended our enriched gene set for HD retrieved with the PSEA analysis (25 genes, Table S1), to include two well-known HD-associated genes from the literature *HTT* [47] and *HSPH1* (Heat shock protein family H (Hsp110) member 1), which has been shown to suppress or disaggregate *HTT* fibrils [48]. This resulted in a set of 27 genes that oscillate significantly in either of the cell lines, which represent about 13% of the total HD-KEEG pathway (hsa05016, 199 genes). In addition to their role in HD, elements from this gene set are also involved in processes such as mitochondrial metabolism, cellular survival and transcriptional regulation.

From the extended HD-related genes list 26 genes were oscillating in SW620 cells: *CASP3*, *CLTC*, *CREB3L4*, *CYCS*, *DLG4*, *GNAQ*, *HIP1*, *HSPH1*, *NDUFA2*, *NDUFA4*, *NDUFA4L2*, *NDUFAB1*, *NDUFS3*, *POLR2C*, *POLR2D*, *POLR2K*, *PPARG*, *SDHD*, *SIN3A*, *SOD2*, *TAF4B*, *TBP*, *TBPL1*, *TP53*, *UQCR10*, and *UQCRHL* (Figure 4D). In contrast, for the primary tumor cell line SW480, only 3 genes (HIP1, HSPH1, HTT) showed significant oscillations (Figure 4C). We observed that among these candidates only HIP1 and HSPH1 depicted significant circadian oscillations in both SW480 and SW620 cells.

**Figure 5:**
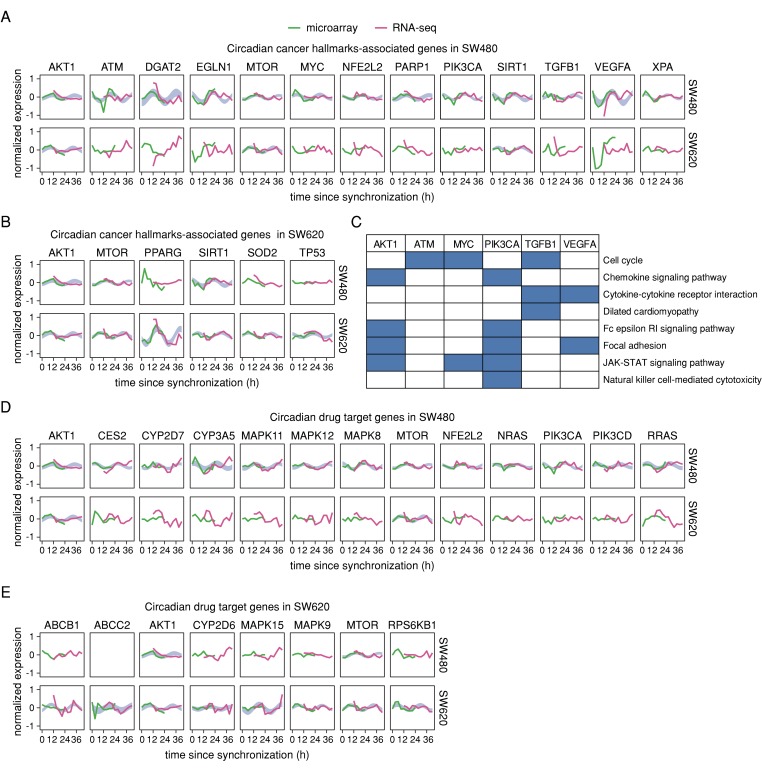
Cancer hallmark-associated and drug target genes show differing oscillatory expression in SW480 and SW620 cells. Normalized time series expression of cancer hallmark-associated genes identified as 24 h rhythmic in (**A**) SW480 and (**B**) SW620 cells based on the concatenated data. (**C**) Phase-clustered circadian KEGG pathways in which cancer hallmark-associated genes are involved in SW480 cells. Normalized time series expression of drug target genes identified as 24 h rhythmic in (**D**) SW480 and (**E**) SW620 cells based on the concatenated data. Microarray expression values are represented by green lines and RNA-seq expression values by pink lines. The respective top row shows the expression in SW480 cells and the respective bottom row in SW620 cells. The blue area marks the confidence area of the harmonic regression fitted to the concatenated data for 24 h rhythmic genes.

We further investigated whether the 24 h rhythmic gene sets of either cell lines included circadian cancer hallmark-associated genes as described by Sulli and colleagues [7] (see also Table S3) and/or circadian drug target genes from Dallmann and colleagues [31]. In SW480 cells, we found 13 cancer hallmarks-associated genes to be 24 h rhythmic: *AKT1*, *ATM*, *DGAT2*, *EGLN1*, *MTOR*, *MYC*, *NFE2L2*, *PARP1*, *SIRT1*, *TGFB1*, *VEGFA*, and *XPA* (Figure 5A). In SW620 cells, we found six genes to be circadian expressed (*AKT1*, *MTOR*, *SIRT1*, *PPARG*, *SOD2*, *TP53*) among the cancer hallmarks-associated set, and three of the genes (*AKT1*, *MTOR*, and *SIRT1*) were also oscillating in SW480 cells (Figure 5B). On the other hand, several of the candidate genes are also part of the phase-clustered pathways identified to be enriched for 24 h rhythmic genes in SW480 cells (Figure 5C). From the list of circadian drug target genes [31], we found 19 drug target genes to be oscillating in either SW480 or SW620 cells (Figure 5D,E), including *AKT1* and *MTOR* that were significantly rhythmic in both cell lines. In SW480 cells, two members of the RAS gene family, *NRAS* and *RRAS*, showed circadian rhythms, whereas circadian drug targets in SW620 cells included the genes *ABCB1* and *ABCC2* that encode for multidrug resistance-associated proteins.

**Figure 6:**
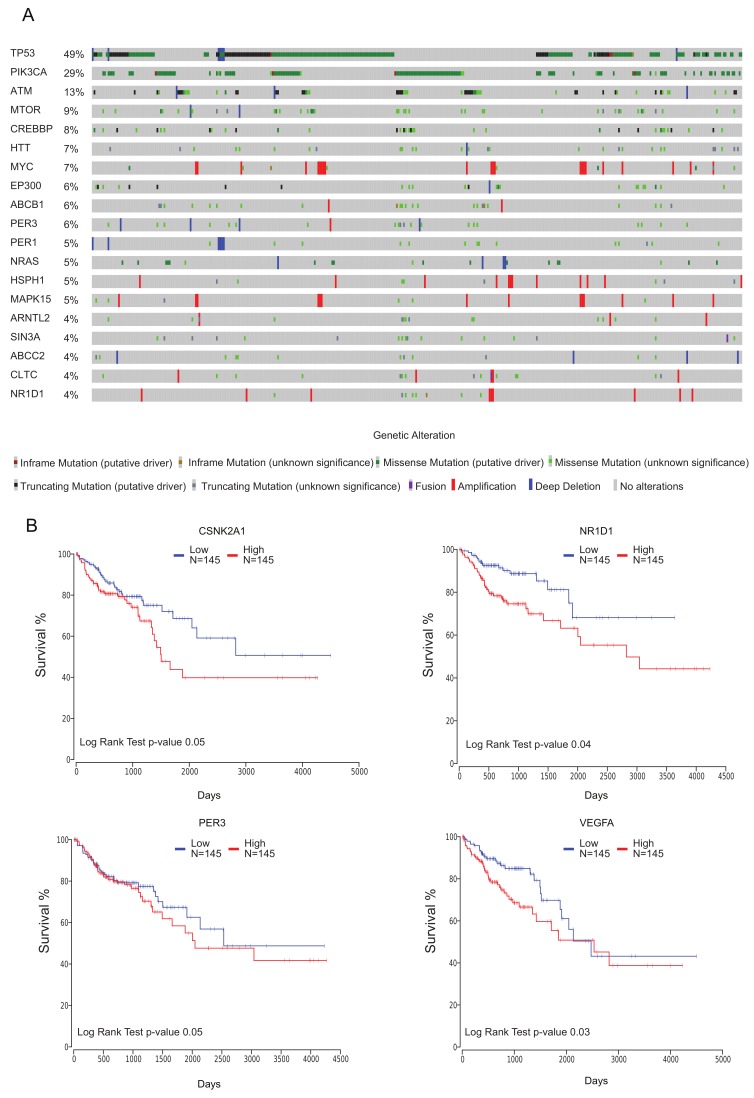
Mutational frequencies and impact of candidate genes on overall survival in TCGA COAD cohort (**A**) Oncoprint graphical summary of alterations in candidate genes that are mutated more than 4% of patients (**B**) Cox-regression based survival curves based on gene expression of candidate genes.

Next, we merged the circadian cancer hallmark-associated gene set (Figure 5A,B) with the list of 27 genes associated with HD curated from PSEA results and literature (Figure 4B,C) and the circadian drug targets (Figure 5D,E) which results in a list of 55 candidate genes (Table S2). Additionally, we included into our analysis elements of the extended core clock network (ECCN) which was previously assembled by our group [32], which were significantly oscillating (in either of the cell lines) within our concatenated data set (Table S4). We analyzed the mutational frequencies from a cohort of colon adenocarcinoma patients [49,50] (439 samples from the PanCancer study) from the TCGA database, as depicted in the OncoPrint graphical summary of our candidate gene set (Figure S4). The top candidate genes with the highest frequency of mutations (cut-off of 4%) were depicted in Figure 6A. Among the most frequently mutated candidates we found genes known to be associated with cancer (*TP53*, *PIK3CA,* and *ATM*), core-clock genes (*PER1, PER3,* and *ARNTL*), and elements of the ECCN (*CREBBP* and *EP300*) (Figure 6A).

Interestingly, we observed that four HD-associated genes, namely *TP53*, *HTT*, *SIN3A*, and *CLTC* were listed among the top frequently mutated candidate genes. In order to assess the impact of the candidate genes on patient survival we performed a differential expression analysis (FDR 0.05, logFC 1.5) using paired tumor and adjacent normal tissue samples (Table S5). The intersection of differentially expressed genes (DEG) and our list of candidate genes, resulted in 12 elements including cancer associated genes (*NRAS*, *MYC,* and *VEGFA*), circadian drug targets (SOD2 and *CES2*) and core-clock and ECCN genes (*AHR*, *CREB1*, *CSNK2A1*, *NFIL*, *NPAS2*, *NR1D1*, and *PER3*) (Table S5). Our analysis showed that the variations in gene expression in some of these genes were significantly associated with patient survival (Figure 6B). In order to access high and low risk groups based on gene expression changes, we selected for each gene the top and bottom thirds of the patient population (*n* = 145 low, *n* = 145 high) as representing high and low expression groups, respectively. The core-clock genes *NR1D1*, *PER3*, *CSNK2A1,* and the cancer associated gene *VEGFA* showed statistically significant results in our Cox-regression based survival plots (log rank test *p* ≤ 0.05).

**Figure 7:**
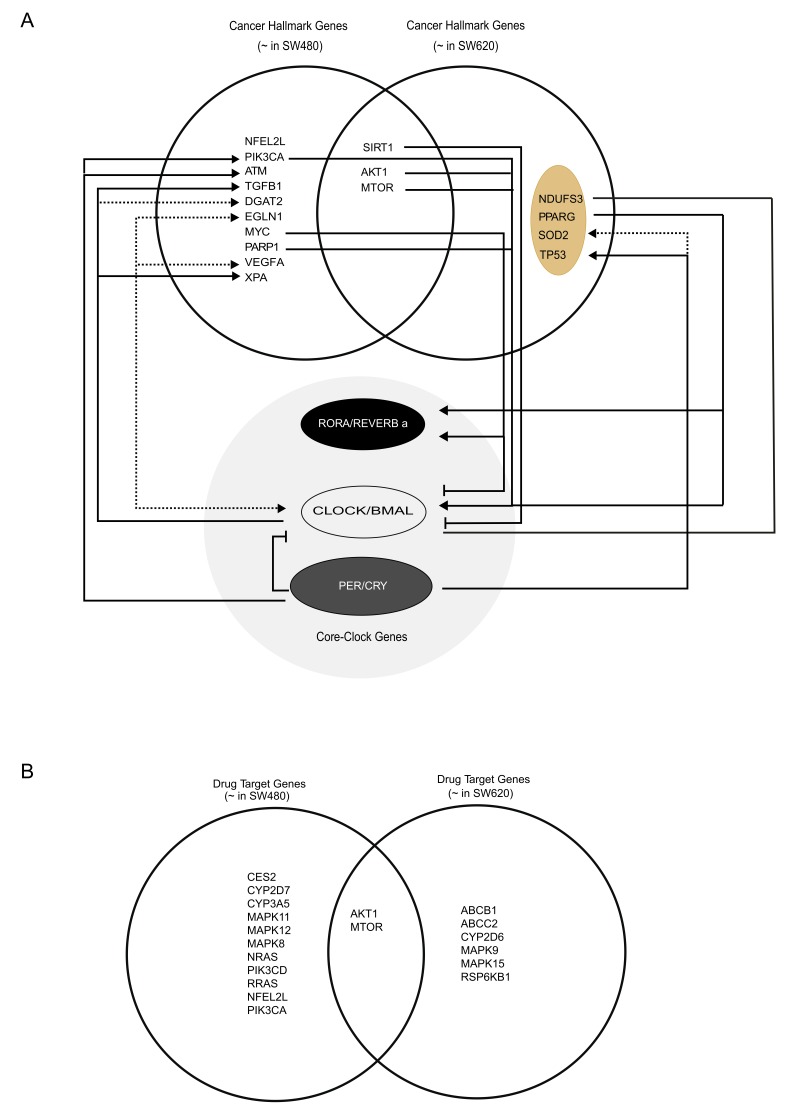
Graphical illustration of molecular interactions between the core-clock network, genes involved in hallmarks of cancer and circadian drug targets. (**A**) Target genes associated with cancer hallmarks and the circadian clock that were found oscillating in analysis. For genes that have multiple route to interact with the clock genes the numbered arrows indicate all potential interaction ways of the target genes to the core clock genes. (**B**) Circadian drug target genes oscillating significantly in analysis. Additionally, some of the candidate cancer associated genes in the diagram were also linked to Huntington’s disease (Table S1). These genes are highlighted with beige and negative regulators of the core circadian clock machinery with black circles. NDUFS3 is indirectly associated with the cancer but dominantly found in HD. “~” stands for significantly oscillating genes and dashed lines indicate the presence of indirect links to the circadian clock machinery.

Although not all core-clock genes were among the most frequently mutated genes within our list, an indirect contribution of the circadian clock on these genes was possible. Thus, we created a diagram including both cancer-associated and circadian drug target genes that were oscillating in our concatenated data sets (Figure 5E). To more precisely illustrate the mechanistic link between these genes and the core-clock genes, we carried out a curated literature search for the interactions between core-clock and cancer-hallmark target genes (Table S3). For SW480 cells, we found a unique set of oscillating genes, namely *NFEL2L*, *PIK3CA*, *ATM*, *TGFB1*, *DGAT2*, *EGLN1*, *MYC*, *PARP1*, *VEGFA*, and *XPA*. For SW620 cells, we found *PPAR-G*, *SOD2,* and *TP53* to be 24 h rhythmic only in this cell line (Figure 7A). *SIRT1, AKT1,* and *MTOR* oscillate in both cell lines in a circadian manner and are associated with the circadian clock and cancer hallmarks (Figure 7A). In addition to the connection to cancer hallmarks and circadian drug targets, some of the circadian genes found in SW620 cells were associated with HD (Figure 4C). We illustrated these HD interaction links in our diagram which included *PPARG*, *SOD2*, and *TP53* (from the work of Sulli and colleagues [7]). and *NDUFS3*, which is indirectly linked to cellular energetics, often dysregulated in cancer (Figure 7A). We further included circadian drug target genes that are oscillating in SW480 and SW620 cells (Figure 7B). As we linked all the interactions between the core-clock genes, cancer hallmarks and circadian drug target genes, we observed that *AKT1* and *MTOR* were circadian expressed, drug target genes, in both cell lines.

## 3. Discussion

CRC is one of the most common types of cancers of the digestive system [51]. One promising new approach in CRC treatment is the time-dependent administration of chemotherapeutic drugs in agreement with the chronotype of the patient [52,53,54]. Accumulating evidence points to an association between malfunctions of the circadian clock and biological events that drive tumorigenesis [6]. Extensive research at the genome-wide level concerning the existence of a putative circadian dysregulation in cancer suggests a role of the core-clock and clock-controlled genes in various biological processes involved in cancer onset and progression [55,56]. However, most of the available data was sampled at a single time point, thereby precluding a detailed assessment of circadian effects. Due to the high cost and effort of producing densely sampled time series data, the meta-analysis of legacy datasets is common in the circadian field. Simulations with synthetic time series have shown that sampling periods shorter than two full circadian cycles can lead to an increase of the false negative rate [57]. Yet, not all circadian datasets fulfil these requirements. Moreover, in recent years, the technical platforms used for gene expression profiling have increasingly shifted from microarrays to RNA-seq. In order to attain more robust results concerning the identity of circadian genes and their rhythmic parameters, e.g., as training data for machine learning approaches [41], it could prove beneficial to extract information from both microarray and RNA-seq legacy datasets.

### 3.1. Cross-Platform Concatenation of Time Series Reveals Differential Circadian Expression in a CRC Model

In the present study, circadian datasets originating from the same CRC cell line model were normalized and concatenated across platforms, yielding a longer time series with two replicate measurements for overlapping time points. For both cell lines, the rhythmicity analysis of the cross-platform dataset resulted in a larger number of 24 h rhythmic genes than for either of the platforms alone. Rhythmic parameters estimated based on the cross-platform time series were well correlated with parameters identified based either on the microarray or the RNA-seq data. Thus, the normalization and concatenation of time series gene expression data from two different platforms is a powerful method to identify circadian genes. With this approach, a higher number of technologically diverse datasets from different platforms can potentially be integrated for meta-analyses. Using the concatenated dataset, we observed several differences between the primary tumor cell line (SW480) and the metastasis-derived cell line (SW620). We identified a differential distribution of gene expression peak times (phases) between the two cell lines. For the primary tumor-derived cells, the distribution of phases was covering nearly the whole circadian day (from 0 to 23.6 h), whereas the enriched pathways in metastasis-derived cells peaked in a relatively narrow time window (4.5 to 10.3 h). Various cancer-associated cellular pathways were enriched for circadian genes in both cell lines, including the cell cycle, DNA replication, DNA damage response, all of which are known to be circadian-regulated. These results obtained in the concatenated data are in line with our previous data based only on the microarray dataset. In addition, the difference in phase distribution between two cell lines indicated a phase shift of 2 to 3 h, which suggested that the circadian clock is dysregulated in the metastatic cell line (SW620). These results are in agreement with previous work from our group [33,39].

### 3.2. Neurodegeneration-Related Pathways Are Clock-Regulated in the Metastatic CRC Cells

We observed several neurodegenerative disease-related pathways in metastasis-derived cells enriched for circadian gene sets, which were not identified for the primary tumor-derived cells. Besides Alzheimer’s disease and Parkinson’s disease, we found a strong association with HD. Neurodegeneration is the main feature of HD, resulting in a triad of motor, cognitive and psychiatric symptoms [58]. In contrast, abnormal uncontrolled cell proliferation is the major characteristic of cancer, placing these two severe diseases at opposite sides of the cell fate spectrum. In line with our findings, previous studies suggested an almost 80% reduction of cancer incidence in HD patients compared to the general population [44,45]. We found 27 HD-associated genes in our cancer cell lines, out of which 26 genes were identified to be oscillating in metastasis-derived SW620 cells and 3 genes were oscillating significantly in primary tumor-derived SW480 cells. The set of HD-associated genes oscillating in SW620 cells included genes involved in cell death (*CASP3*, *TP53*, *CYSC*), mitochondrial oxidative phosphorylation (*SOD2*, *SDHD*, *NDUFA2*, *NDUFA4*, *NDUFA4L2*, *NDUFAB1*, *NDUFS3*, *UQCR10,* and *UQCRHL*), and gene transcription by RNA polymerase II (*POLR2A*, *POLR2C*, *POLR2K*, *TAFA4*, *TBP*, and *TBPL1*). 

*HTT* is expressed throughout the body and the mutant protein not only affects the brain but also peripheral tissues as weight loss, cardiomyopathies and skeletal muscle malfunction have been described for HD [59]. Interestingly, cancer has been reported to account only for 5% of the leading causes for death in HD [45]. However, mutant *HTT* can also contribute to the severity and progression of a specific cancer type. For example, while *HTT* regulates the normal development of the mammary tissue [59,60], mutant *HTT* expression is pro-metastatic in breast cancer and can accelerate tumor development through ErbB2/HER2 signaling [61]. Notably, *mHTT* has been reported to interact directly with *SIRT1*, a clock gene, and inhibit its activity [62]. It is interesting to note that we found significantly more circadian regulated genes in metastatic CRC cells than in primary tumor-derived cells. Interestingly, *MLH1* has been identified as a modifier of HD onset [63] and has also been associated with higher risk of colorectal cancer [64,65]. In particular, *MLH1* plays an important role in the microsatellite instable biological subtype of colorectal cancer [66]. Microsatellite instability (MSI) shows different pathological characteristics, as well as poor response to traditional chemotherapeutic reagents. Surprisingly, we found that *HTT* oscillated in SW480 cells, but not in SW620 cells, although its interacting factor, *HIP1,* oscillated in both cell lines. *SOD2* encodes for a protein involved in reactive oxygen species clearance in mitochondria, and oscillates in SW620 cells. *SOD2* has a rhythmic acetylation that is suppressed in mice with Clock mutation [67]. Remarkably, five genes of the *NDUF* family that encodes for mitochondrial enzymes involved in oxidative phosphorylation oscillate in SW620 cells, but not in SW480 cells. One of them is *NDUFS3*, a potential biomarker to discriminate invasive from normal breast cancer [68], whose expression is controlled by the CLOCK gene [69].

### 3.3. Circadian Regulation of Cancer Hallmarks and Molecular Drug Targets

Perturbations of core-clock genes have been reported to influence the hallmarks of cancer in different cancer models including the CRC cell line model investigated in this manuscript. Differential effects on the cell cycle were reported to be associated to the expression levels of the core-clock gene *BMAL1* [27]. The downregulation of *BMAL1* in SW480 cells lead to an increase of cells in the S-phase and a decrease of cells in the G1-phase, in SW620 cells—where *BMAL1* expression is already very low no significant effect was observed. Thus, decreased expression levels of *BMAL1* seems to be associated with more proliferative scenarios in these CRC cell lines. Moreover, *BMAL1* knockdown impacts proliferation, and apoptosis in a time-dependent manner [39]. Upon knockdown of *BMAL1*, SW480 cells proliferate faster than the corresponding control cells and their proliferation profile resembles that of SW620 cells, leading to the assumption that a dysregulated clock promotes a more metastatic phenotype. The effect of *BMAL1* knockdown on cell viability was shown to be time-dependent for both cell lines, with an increased viability observed for later time points, with more prominent changes in SW480 cells. The downregulation of *BMAL1* led to significantly lower apoptosis rates for both SW480 and SW620 cells, which is in line with the assumption of the clock acting as a tumor suppressor [70]. This circadian regulation of cellular processes impacts the time of day of drug administration (chronotherapeutics), the distribution, metabolism, and excretion of therapeutic agents, and is used to improve the efficiency of anti-cancer drugs while reducing undesired side effects [71]. The genes that were oscillating in our analysis are critical elements of pathways that are dysregulated in various types of cancer [72]. Therefore, targeting these genes has been the scope of development of new and more effective cancer drugs [73]. The link we observe to these genes in our data analysis reveal interesting candidates for the optimization of cancer treatment. In particular, to link the oscillating genes that are known to be targets of cancer drugs [7,31], to the hallmarks of cancer-related genes and ECCN genes [32], we investigated the role of our candidate genes in TCGA colon adenocarcinoma samples. Our analysis of mutational frequencies revealed core-clock genes including *PER1, PER3, ARNTL2,* and *NR1D1* and two ECCN genes *CREBBP* and *EP300* to be mutated in more than 4% of the patients. We further performed a differential gene expression analysis between paired tumor and normal samples which revealed 12 of our candidate genes to be differentially expressed. From those, *NR1D1*, *CSNK2A1*, *PER3*, and *VEGFA* showed significant correlation with patient survival. This indicates that refinement of cancer treatments based on the individual circadian rhythm may provide additional benefits for the therapy outcome. Although not all of the core-clock genes showed high mutational frequencies within the TCGA cohort, we did not exclude possible indirect contributions of the circadian clock on the candidate genes. Therefore, we illustrated the connection of these cancer associated and circadian drug target genes to the core-clock machinery based on the literature. The intersection between cancer-associated and circadian genes in both cell lines resulted in three genes, *SIRT1*, *AKT1*, and *mTOR. SIRT1* is a well-characterized circadian regulated member of Sirtuins family involved in histone and/or protein deacetylation [74], and plays an important role in various cancer related processes such as inhibition of apoptosis for cell survival and oxidative damage or DNA damage induced senescence.

One of the interesting candidate genes identified in our study—Akt1 is both a cancer hallmark and a relevant circadian drug target gene. Interestingly, the activity of Akt1 was found to depict circadian rhythms due to periodic fluctuations in its phosphorylation [75]. Akt circadian activity might also influence other pro-tumorigenic processes such as cellular proliferation and promotion of cell survival. In the same study, the authors have shown that acute depletion of Cdk2, as well as deletion of Cyclin A2 or Cdk2 resulted in the decrease of Akt phosphorylation and subsequently its activity, and suggested that variation in Cyclin A2 may partially explain the pro-tumorigenic activity of Akt [75]. It would be interesting in future work to investigate this mechanism in the context of colorectal cancer.

Previous studies by Sessa and colleagues showed the crucial role of AKT on proper functioning of the CLOCK by phosphorylation from a specific serine site [76]. More recently, an experimental jet lag protocol aimed to test the role of chrono-modulated drug delivery for Cdk4/6 inhibitors in mice revealed a significant upregulation of *AKT1* [77]. It is also well known that AKT can influence the circadian phenotype both via the upstream (PI3K) and downstream substrates such as the transcription factor FOXO [78]. However, to our knowledge, the potential benefit of chrono-modulation for AKT targeting has not been studied in the context of colorectal cancer. Also the inhibition of mTOR, as a result of hypoxia related acidification of the cellular microenvironment, was found to lead to clock dysfunction [79]. The mTOR pathway consists of a signaling cascade that serves various crucial cellular regulatory functions such as metabolism, proliferation, and growth [80]. Accumulating evidence points to the role of the mTOR pathway in the regulation of the circadian system [81,82,83], as well as in various pathologies including cancer [84]. A study with Everolismus, an mTOR inhibitor, showed a better antitumor efficiency in mice when administered at ZT12.

Next, we aimed to identify the implication of our findings for molecular drug targets. Our results showed that two well-known drug target genes in cancer therapy, *AKT1* and *mTOR* were oscillating in both CRC cell lines. Ideal timing is a crucial but underestimated trait in drug development and the genes that we found to be oscillating in both cell lines are critical elements of frequently dysregulated pathways in various types of cancer [72]. Therefore, targeting these genes is one of the aims in the development of new and more effective cancer drugs [73]. The link we observe between the circadian clock and these genes, in our data analysis, makes them interesting candidates for the time of day optimization of cancer treatment. In addition to these common drug target pathways, we identified unique sets of drug target genes in our analysis including members of the RAS family. NRAS and RRAS, exhibit circadian rhythmicity in SW480 cells. RAS can activate cell cycle regulators such as the cyclin dependent kinase inhibitor p16 (Ink4a), which results in dysregulation of the cell cycle. RAS induced activation of Ink4a leads to cell cycle arrest and acts as a tumor-suppressive mechanism [85]. In addition, perturbations in oncogenic RAS were shown to modulate the circadian clock in an in vitro CRC model [86]. In a follow-up study with mouse embryonic fibroblast, the authors found a role for the tumor-suppressors Ink4a and Arf as mediators of RAS induced changes on the circadian phenotype, which promoted a more cancer-prone scenario in these cells, by enhancing proliferation [27]. In our current results, NRAS and RRAS exhibit differential circadian expression in SW480 cells and the metastasis derived cells (SW620). Further experimental validation is required, to investigate whether the difference observes in the oscillatory behavior of these two target genes, as a result from the circadian phenotypes of both cells, might have an implication in the tumorigenic properties of these CRC cells and to which extent are alterations of circadian regulation a putative driver of tumor progression.

In the metastasis-derived SW620 cells, two members of the ABC transporter superfamily were found to be oscillating. The overexpression of ABC transporters is known to be involved in multiple drug resistance which leads to therapy resistance. Interestingly, independently from our dataset, *ABCB1* was among the top candidate genes in our mutation frequency analysis based on TCGA data. In recent years, it has been reported that tyrosine kinase inhibitors also repress ABC transporters [87,88]. There are various receptor tyrosine kinase inhibitors used in the clinics, some of which have already been studied for the enhancement of therapeutic response by chrono-modulated fashion. For example, Erlotinib which is an inhibitor of Ras-Raf-MAPK receptor tyrosine kinase activity was found to be more effective at ZT1 in contrast to ZT13 in female mice (ZT, Zeitgeber Time is defined as the time of external or environmental cues that entrain or synchronize an organism’s circadian clock) [89,90]. Another study showed the enhanced impact of lapatinib, a dual inhibitor of EGFR and Ras-Raf-MAPK cascade, in male mice when administered at ZT23, resulting in better tumorigenesis inhibition and angiogenesis compared to ZT13 [31]. Given the results of these previous studies and circadian transcription of two members of ABC transporters in our metastatic cell line, treatment outcome with receptor tyrosine kinases could be potentially advanced based on the patient specific circadian rhythm. This might be a potential solution for a frequent problem in clinics: Therapy resistance and relapse. It should be also taken into account that the treatment outcome is not solely dependent on the individual chronotype, but also on additional traits such as age and gender [91]. Therefore, new methodologies should be developed to study various traits in cancer development within currently available data. 

## 4. Materials and Methods

### 4.1. Microarray Data Pre-Processing

Raw microarray expression data of the human CRC cell lines SW480 and SW620 from El-Athman, Fuhr and Relógio [33] was downloaded from the ArrayExpress database (E-MTAB-5876). The data was pre-processed for all time points of each dataset as one batch using the RMA methodology [92,93] as implemented in the oligo package (v1.42.0) [94]. Transcript clusters were annotated with Ensembl IDs using Affymetrix HTA 2.0 annotation data (hta20transcriptcluster.db, v8.7.0). For genes annotated by multiple transcript clusters, the transcript cluster with the highest mean expression over all time points of the respective cell line was chosen to represent gene-level expression.

### 4.2. RNA-seq Data Pre-Processing

Raw RNA-seq data of the human CRC cell lines SW480 and SW620 from El-Athman, Knezevic, Fuhr and Relógio [34] was downloaded from the ArrayExpress database (E-MTAB-7779). Quality control of the 75 bp paired-end reads was performed using FastQC (v0.11.7) [95] and adapter sequences were cut using Trimmotatic (v0.38) [96] with TruSeq3-PE-2 adapter sequences. Only paired-end reads were retained. Alignment and quantification of the RNA-seq data was conducted in a tripartite pipeline that makes use of two prominent tools for the alignment and quantification of RNA-seq data, STAR (v2.6.0a) [97] and Salmon (v0.10.2) [98], and a combination of both. In the *STAR + featureCounts* pipeline, sequencing reads were aligned to the human genome (Homo_sapiens.GRCh38, Ensembl release 92) using STAR with default parameters. Aligned reads were then assigned to genomic features and quantified using featureCounts [99] as implemented in the R package Rsubread (v1.34.4) [100]. In the *STAR + Salmon* pipeline, the genome alignment was translated to transcript coordinates using STAR with the option—quantMode TranscriptomeSAM. Reads were quantified based on the STAR transcriptome alignment using Salmon in alignment-based mode with default parameters and the—seqBias option. In the Salmon pipeline, reads were directly quantified using Salmon in mapping-based mode with default parameters and the—seqBias option. The transcriptome indices for Salmon were built based on Homo_sapiens. GRCh38.cdna, Ensembl release 92. For both the *STAR* + Salmon and the Salmon pipeline, the resulting transcripts per million (TPM) count tables were scaled using the tximport package (v1.6.0) [101] by first multiplying TPM by feature length and then scaling up to the library size (lengthScaledTPM), resulting in summarized gene-level (txOut = FALSE, based on Ensembl Transcript IDs) count estimates. For all three RNA-seq pipelines, counts were log_2_-transformed using the cpm function and applying the TMM method from the R package edgeR (v3.20.9) [102]. Only genes with at least 0.5 CPM on average over all time points of a cell line were retained and counts were renormalized using only the selected genes.

### 4.3. Cross-Platform Normalization and Concatenation of Time Series

To enable a cross-platform concatenation of the time series gene expression data, we applied Training Distribution Matching (TDM) as implemented in the R package TDM (v0.3) [41]. TDM has originally been developed for making machine learning applications trained on legacy microarray data applicable to RNA-seq data. We used the TDM algorithm to transform the RNA-seq data for each cell line individually, thereby making its distribution comparable to that of the microarray data of the same cell line, while keeping inter-observation dependencies of the RNA-seq time series intact. A longer time series (0 to 42 h after synchronization) was produced by first individually normalizing both the microarray and the TDM-transformed RNA-seq data to the mean of the shared time points (12 to 24 h after synchronization) and then concatenating both datasets. Values of the overlapping time points were treated as replicate measurements.

### 4.4. Rhythmicity Analysis

For the detection of circadian genes and their parameters (phase and amplitude), the harmonic regression method was applied as implemented in the R package HarmonicRegression (v1.91) [103], using the robust option and setting the period to 24 h. For both the microarray and RNA-seq data, original (not log_2_-scaled and not TDM-transformed) expression values of commonly expressed genes between the platform were used as input. For the concatenated data, the normalized and TDM-transformed time series were used. Harmonic regression *p*-values were Benjamini-Hochberg adjusted for multiple testing. Statistical significance for 24 h rhythmic genes was set at *q* < 0.05 and a relative amplitude ≥ 0.1.

### 4.5. Correlation Analysis

For each cell line, correlation of expression values of commonly expressed genes was conducted between all four pipeline methods (microarray, STAR + featureCounts, STAR + Salmon, Salmon) in a pairwise manner for samples taken at identical time points, considering only those time points that are shared between all methods (12–24 h). The resulting Pearson correlation coefficients were averaged over all time points for each method comparison. Circadian parameters (i.e., phases and relative amplitudes) of 24 h rhythmic genes were compared between the three datasets (microarray, RNA-seq, and the concatenation of both) in a pairwise manner. Only genes that were commonly identified to be 24 h rhythmic were considered for the correlation analyses. Circular Pearson correlation coefficients were computed for phases of 24-h rhythmic genes and statistical significance was tested using the function core-circular from the R package circular (v0.4-93) for circular statistics [104]. For relative amplitudes, Pearson correlation coefficients were computed and statistical significance was tested using the function cor.test from the R package stats.

### 4.6. Functional Enrichment

Significantly phase-clustered circadian pathways enriched for the 24 h rhythmic genes sets were detected based on the concatenated dataset were identified by Phase Set Enrichment Analysis (PSEA) [105]. Phases of 24 h rhythmic genes were rounded to the full hour. Gene sets for KEGG pathways (c2.cp.kegg.v6.2) were downloaded from the Molecular Signatures database (MSigDB) [106]. The Kuiper test was used to identify circadian pathways against a uniform background distribution. Pathways containing less than five 24 h rhythmic genes were excluded and statistical significance was set at *q* < 0.05.

### 4.7. Analysis of CRC Data From a Cohort of Patients Retrieved from the TCGA Data Base

Clinical information for colon adenocarcinoma with overall patient survival obtained from The Cancer Genome Atlas (TCGA) research network established by the National Cancer Institute at the National Institute of Health (available at https://portal.gdc.cancer.gov). Mutational frequencies in the TCGA COAD patient population (using 439 samples from PanCancer Atlas all colon adenocarcinoma data) were plotted using cBio Cancer Genomics Portal (available at http://cbioportal.org) developed by Memorial Sloan-Kettering Cancer Center [107,108]. Oncoprint functionality used for graphical representation of mutation frequency summary plots for total number of 92 target genes derived from our analysis. These included 55 candidate genes curated from our analysis and 39 genes from reduced Extended Core Clock Network that include genes that oscillate significantly in either of the cell lines within our concatenated data. When the gene lists were intersected, 2 genes were common in between ECCN and disease candidate genes, which resulted in 92 unique candidate genes. The differential gene expression analysis performed using R Studio Bioconductor package TCGAbiolinks [109,110] package v.2.15.3. FPKM-UQ normalized transcriptome profiling with gene expression quantification values were downloaded for COAD patients with 439 tumor and 41 normal tissue samples obtained from the adjacent normal tissue of the patients. The data normalized based on quantile method and the differential expression analysis performed using EdgeR pipeline, which follows a generalized linear models (glmLRT) functionality. We restricted our analysis for tumor and normal samples that show a fold change (FC) greater than 1.5 and FDR < 0.05 and regarded the genes pass this cut-off as differentially expressed. The resulted DE genes were then intersected with our candidate genes list which resulted in 12 common genes. For these genes we plotted survival curves using a Cox model that includes coxph (Surv(times,died) ~gene + age) via OncoLnc (available at http://www.oncolnc.org) [111]. The survival data being used in the interactive portal includes clinical data for only patients who contain all the clinical information needed for the analysis and based on a follow up or survival days greater than zero. Previous studies revealed contribution of additional clinical parameters to the benefit provided by chronotherapy (i.e., age and sex). Therefore, in our cox survival analysis we added patient age as a clinical parameter in addition to gene expression. The patient cohort for the gene expression level survival plots selected for Colon Adenocarcinoma (COAD) and the expression change were compared between the top third (33%) and bottom third (33%) of the total dataset which allows exclusion of overlapping data slices. Based on the mean expression value of the candidate gene, the change in gene expression levels were categorized as high and low for equal number of patients in the cohort (*n* = 145 for high expression group and *n* = 145 for low expression group).

## 5. Conclusions

The circadian clock can directly or indirectly regulate and interact with biological events that are likely to play a role in tumor initiation and progression [112]. These include circadian regulation of the cell division cycle [27], DNA damage repair [18], metabolism [11], apoptosis, and redox events. A link between the circadian clock and the hallmarks of cancer has emerged in recent years [6,7,113]. 

Colorectal cancer is one of the best studied examples for administration of chrono-modulated chemotherapeutic. Multiple clinical studies showed therapeutic benefits of chronotherapy in CRC patients with conventional chemotherapeutic reagents such as oxaliplatin [114], irinotecan [115], 5-fluorouracil (5-FU) [116], folinic acid [116] and leucovorin [117], which revealed differential contribution of additional clinical parameters to the benefit provided by chronotherapy (i.e., age and sex). Therefore, in our study we aimed to investigate circadian (dys-)regulation in a CRC cellular model at the transcriptome level. We used two colorectal cell lines derived from the primary tumor cells and metastasis of the same patient to study circadian properties from time course datasets, including arrays and sequencing data which were merged and concatenated to generate a data set with a longer time courses and partly replicated time points. Our approach can be useful for other time series data sets obtained from different high-throughput platforms. 

We observed differentially enriched pathways in SW480 and SW620 cell lines including multiple neurodegenerative disease-related pathways in the metastasis-derived SW620 cells. We focused on HD and found 25 HD- associated genes to be circadian expressed in the metastasis cells. We further investigated the indirect link between these cancer hallmarks genes and circadian drug targets in our datasets and identified *AKT1* and *MTOR* as potential circadian drug targets for both of our cell lines.

Analyzing gene expression data from a cohort of 439 patients with CRC available in the TCGA Pan-Cancer colon adenocarcinoma (COAD) study, we strengthened the connection found between cancer and HD.

Our study, using a cross-platform concatenation of existing circadian data sets, provides a comprehensive analysis of circadian properties at different stages of colorectal cancer and highlights their potential impact in a large patient dataset. Further in vitro and in vivo investigation of circadian disruption and its impact in the time-dependent regulation of the target genes identified in our analysis, including the functional outcomes of such perturbations on cell cycle, proliferation and apoptosis, may contribute to design individualized cancer treatment regimens, which consider the individual circadian rhythm of patients.

## Supplementary Materials

The following are available online at https://www.mdpi.com/2072-6694/12/4/963/s1, Figure S1: Mapping rates and number of expressed genes for different methods and cut-offs, Figure S2: RNA-seq expression data before and after cross-platform normalization, Figure S3: Comparison of circadian parameters between sets of 24 h rhythmic genes identified for different methods, Figure S4: OncoPrint mutational frequency summary all candidate genes (including cancer hallmark associated genes, circadian drug targets, HD and ECCN) in TCGA pan-cancer colon adenocarcinoma study, Figure S5: Cox regression-based survival curves for 12 genes found common in differentially expressed genes analysis and our candidate genes list, Table S1: List of Genes Identified from PSEA for HD, Table S2: List of 55 genes found to be HD-related, cancer hallmark genes that are associated with the circadian clock and circadian drug target genes, Table S3: Literature references for Figure 7A, Table S4: The list of genes included in the Extended Core Clock Network, Table S5: Differentially expressed genes (DEG) between tumor and adjacent normal tissue samples (FC > 1.5 and FDR < 0.01).
