# Supplementary material for: Analysis of the Circadian Regulation of Cancer Hallmarks by a Cross-Platform Study of Colorectal Cancer Time-Series Data Reveals an Association with Genes Involved in Huntington’s Disease
Source: Cancers (Basel). 2020 Apr 13;12(4):963. doi: 10.3390/cancers12040963 (PMC7226183; doi:10.3390/cancers12040963)
Supplement: Supplementary file 1 [file cancers-12-00963-s001.zip › Supplementary_Material/Figure S4.pdf]

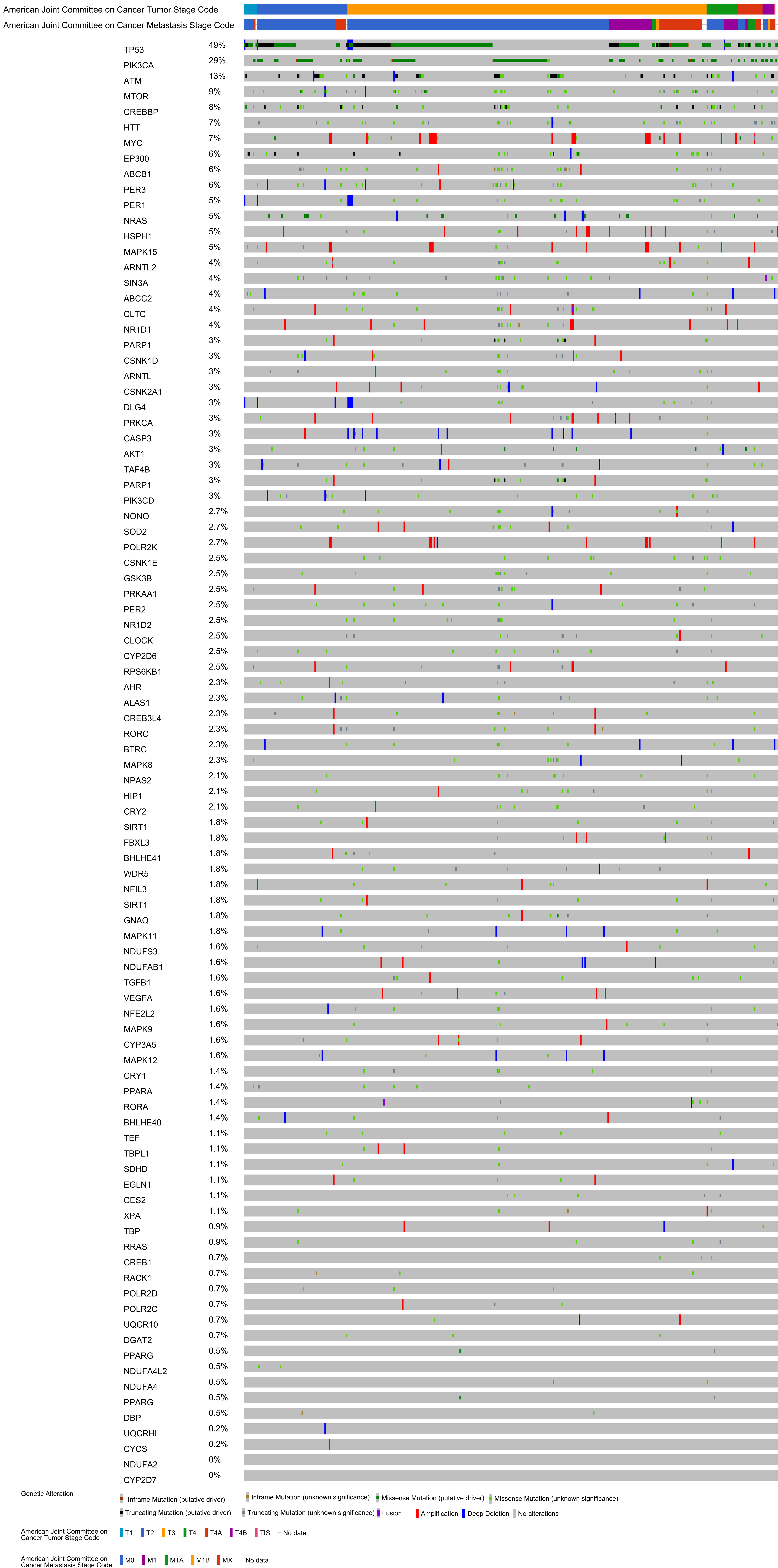
American Joint Committee on Cancer Tumor Stage Code
American Joint Committee on Cancer Metastasis Stage Code
TP53 49%
PIK3CA 29%
ATM 13%
MTOR 9%
CREBBP 8%
HTT 7%
MYC 7%
EP300 6%
ABCB1 6%
PER3 6%
PER1 5%
NRAS 5%
HSPH1 5%
MAPK15 5%
ARNTL2 4%
SIN3A 4%
ABCC2 4%
CLTC 4%
NR1D1 4%
PARP1 3%
CSNK1D 3%
ARNTL 3%
CSNK2A1 3%
DLG4 3%
PRKCA 3%
CASP3 3%
AKT1 3%
TAF4B 3%
PARP1 3%
PIK3CD 3%
NONO 2.7%
SOD2 2.7%
POLR2K 2.7%
CSNK1E 2.5%
GSK3B 2.5%
PRKAA1 2.5%
PER2 2.5%
NR1D2 2.5%
CLOCK 2.5%
CYP2D6 2.5%
RPS6KB1 2.5%
AHR 2.3%
ALAS1 2.3%
CREB3L4 2.3%
RORC 2.3%
BTRC 2.3%
MAPK8 2.3%
NPAS2 2.1%
HIP1 2.1%
CRY2 2.1%
SIRT1 1.8%
FBXL3 1.8%
BHLHE41 1.8%
WDR5 1.8%
NFIL3 1.8%
SIRT1 1.8%
GNAQ 1.8%
MAPK11 1.8%
NDUFS3 1.6%
NDUFAB1 1.6%
TGFB1 1.6%
VEGFA 1.6%
NFE2L2 1.6%
MAPK9 1.6%
CYP3A5 1.6%
MAPK12 1.6%
CRY1 1.4%
PPARA 1.4%
RORA 1.4%
BHLHE40 1.4%
TEF 1.1%
TBPL1 1.1%
SDHD 1.1%
EGLN1 1.1%
CES2 1.1%
XPA 1.1%
TBP 0.9%
RRAS 0.9%
CREB1 0.7%
RACK1 0.7%
POLR2D 0.7%
POLR2C 0.7%
UQCR10 0.7%
DGAT2 0.7%
PPARG 0.5%
NDUFA4L2 0.5%
NDUFA4 0.5%
PPARG 0.5%
DBP 0.5%
UQCRHL 0.2%
CYCS 0.2%
NDUFA2 0%
CYP2D7 0%
Genetic Alteration
Inframe Mutation (putative driver)
Inframe Mutation (unknown significance)
Missense Mutation (putative driver)
Missense Mutation (unknown significance)
Truncating Mutation (putative driver)
Truncating Mutation (unknown significance)
Fusion
Amplification
Deep Deletion
No alterations
American Joint Committee on Cancer Tumor Stage Code
T1 T2 T3 T4 T4A T4B TIS No data
American Joint Committee on Cancer Metastasis Stage Code
M0 M1 M1A M1B MX No data
