# Supplementary figures and images for: Analysis of the Circadian Regulation of Cancer Hallmarks by a Cross-Platform Study of Colorectal Cancer Time-Series Data Reveals an Association with Genes Involved in Huntington’s Disease
Source: Cancers (Basel). 2020 Apr 13;12(4):963. doi: 10.3390/cancers12040963 (PMC7226183; doi:10.3390/cancers12040963)

A

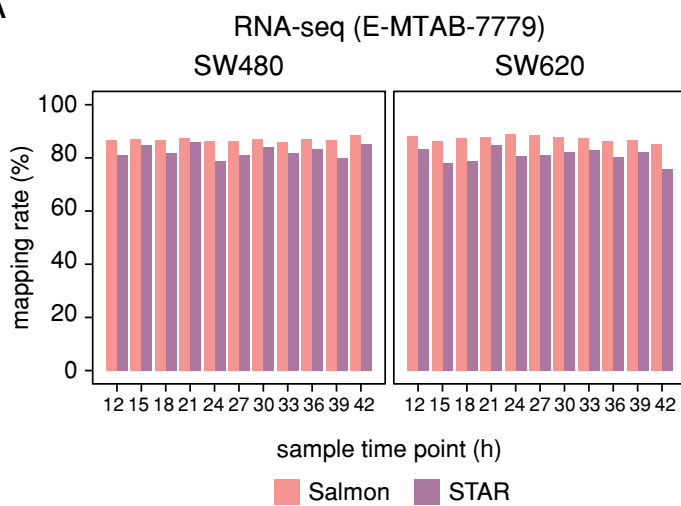

B

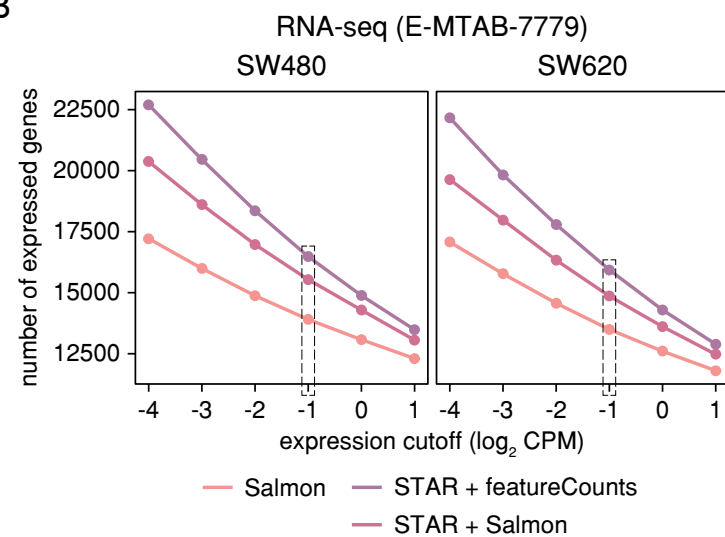

C

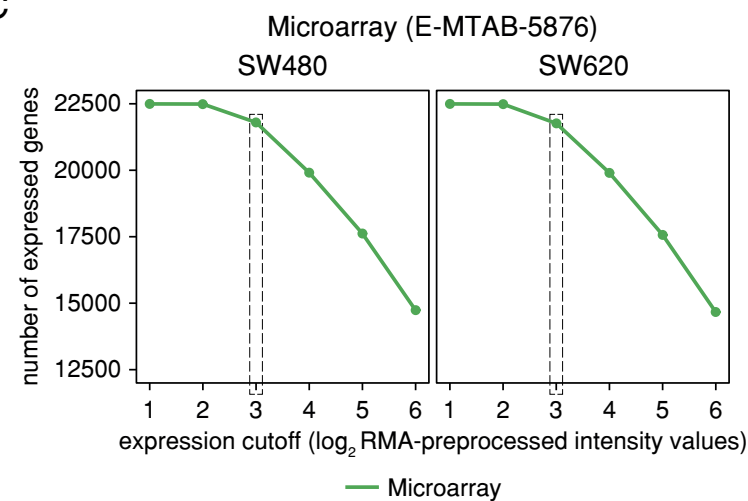

Supplement: Supplementary file 1 [file cancers-12-00963-s001.zip › Supplementary_Material/Figure S1.pdf]

A

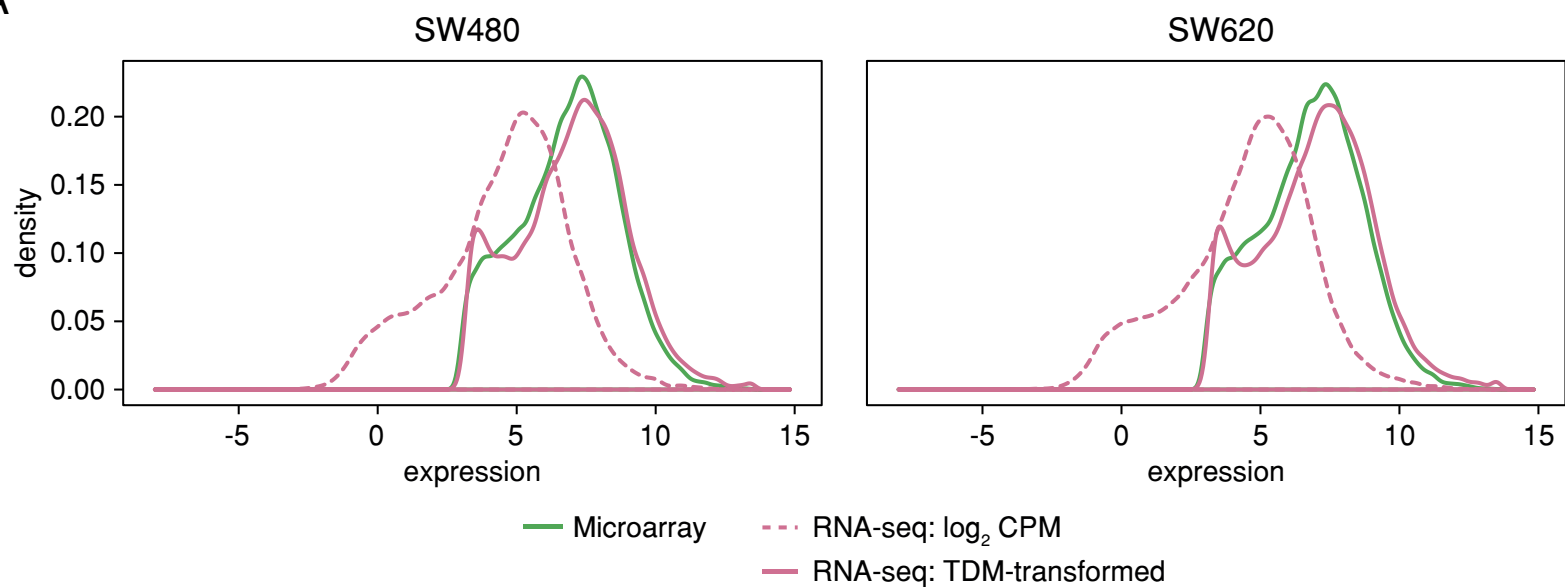

B

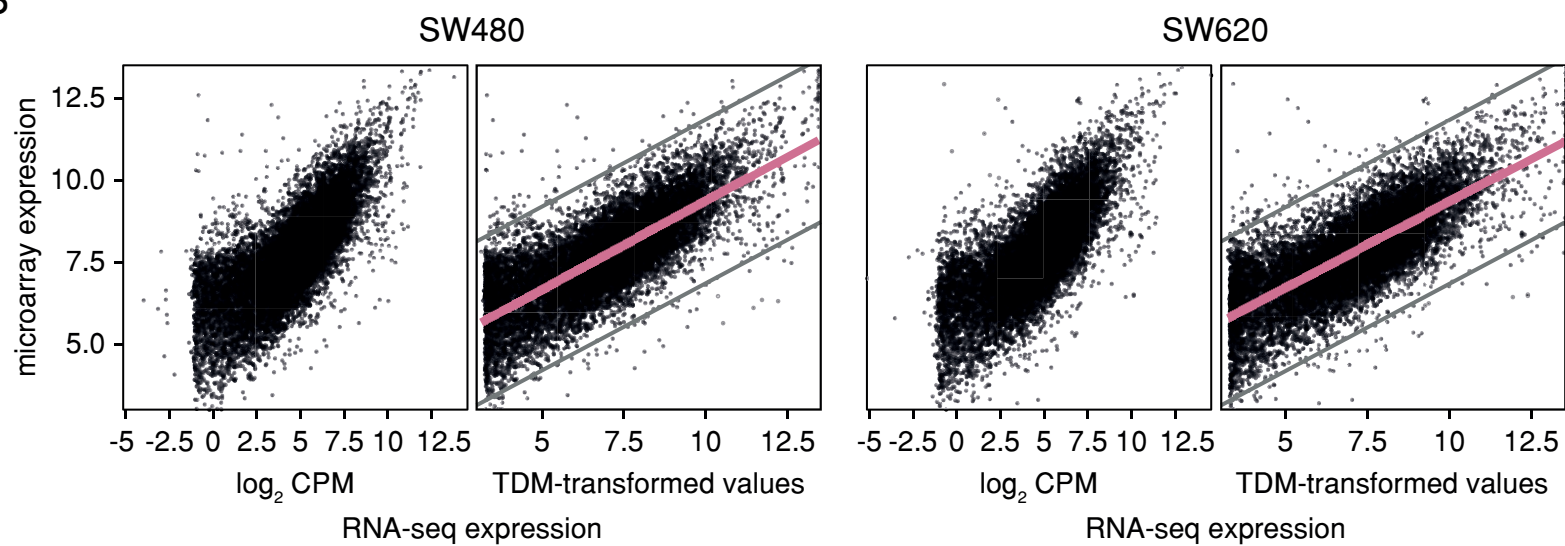

Supplement: Supplementary file 1 [file cancers-12-00963-s001.zip › Supplementary_Material/Figure S2.pdf]

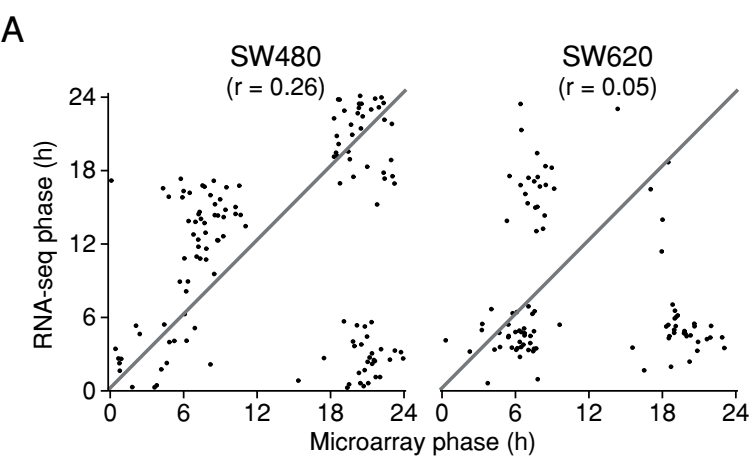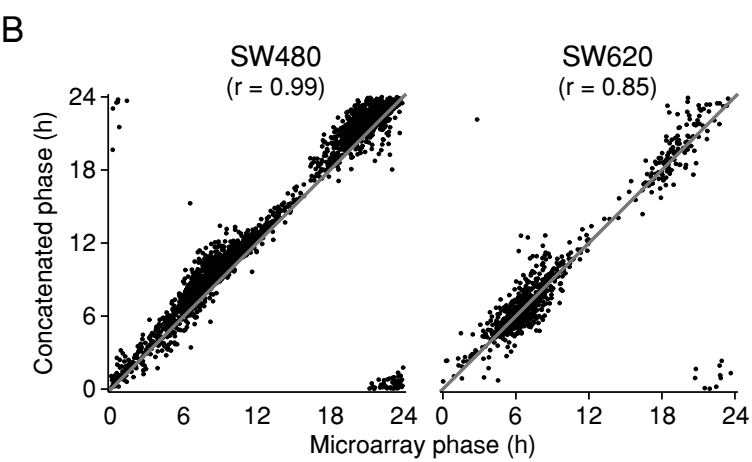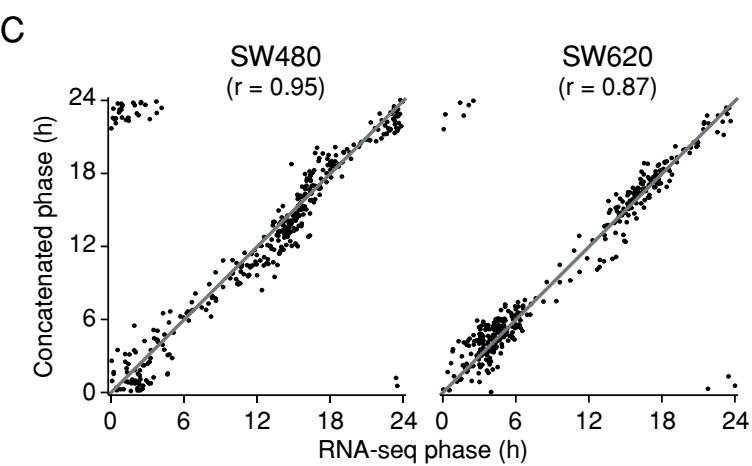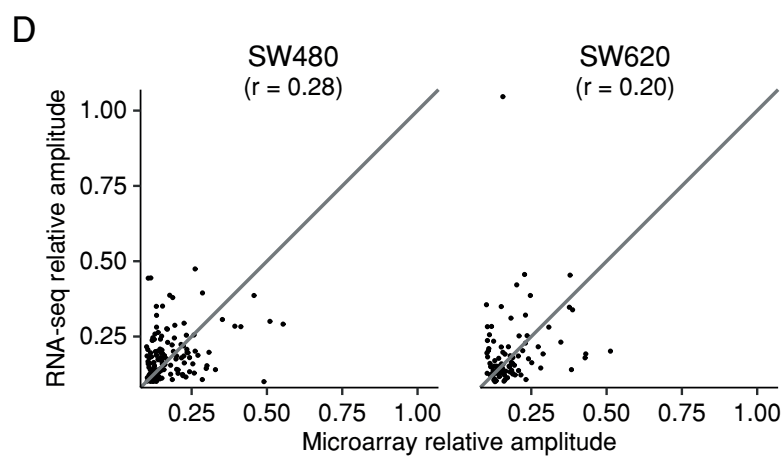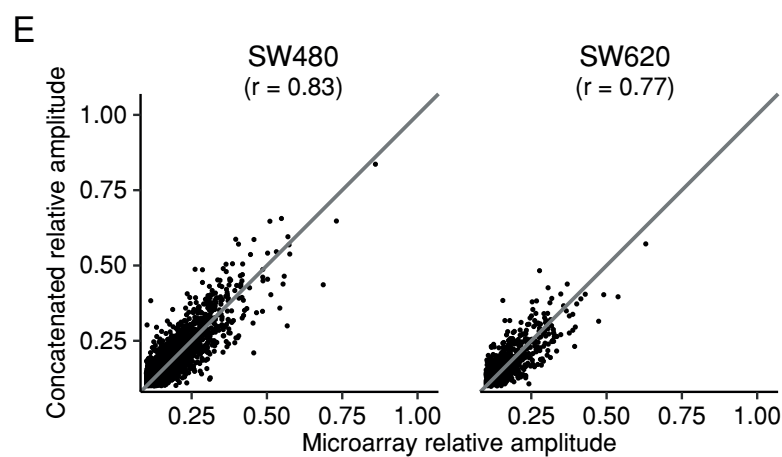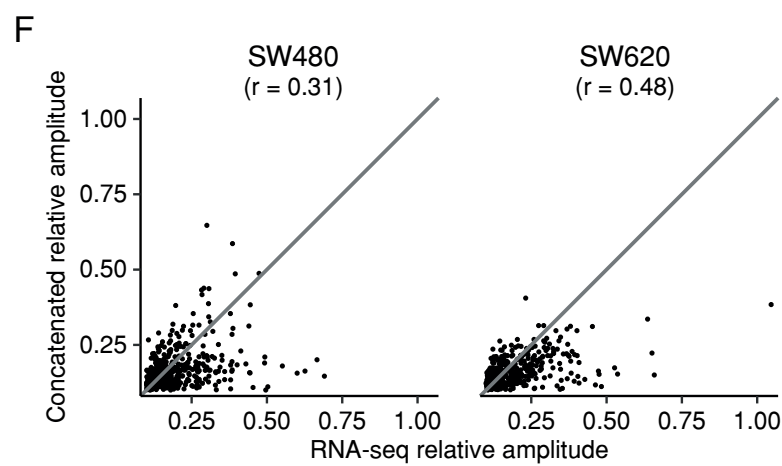

Supplement: Supplementary file 1 [file cancers-12-00963-s001.zip › Supplementary_Material/Figure S3.pdf]

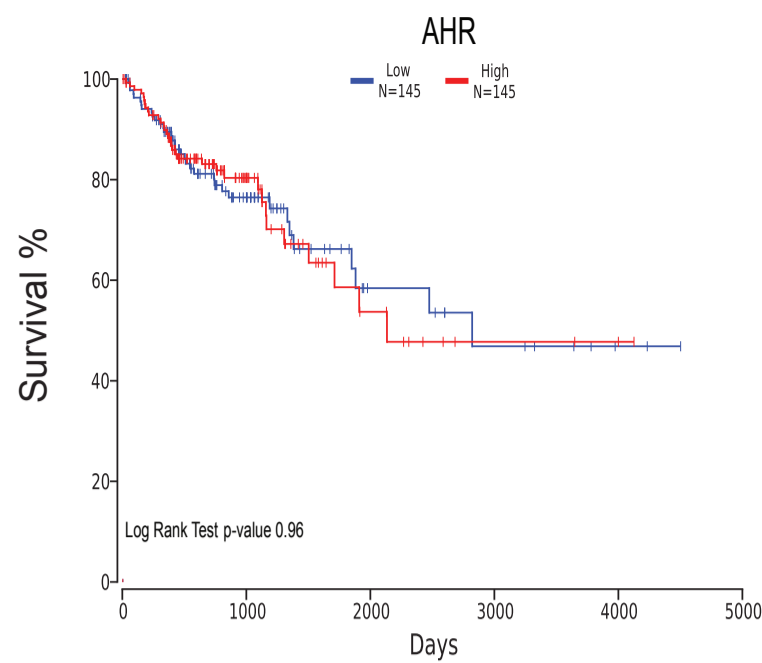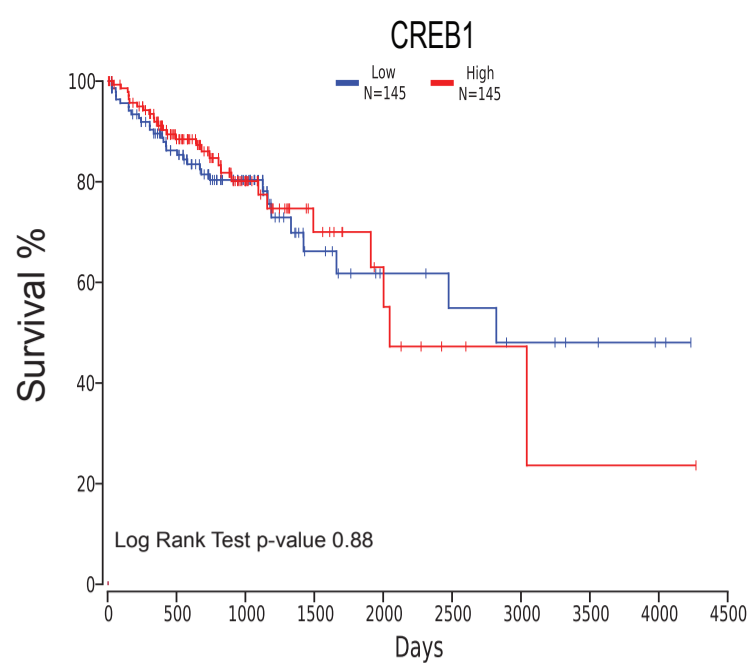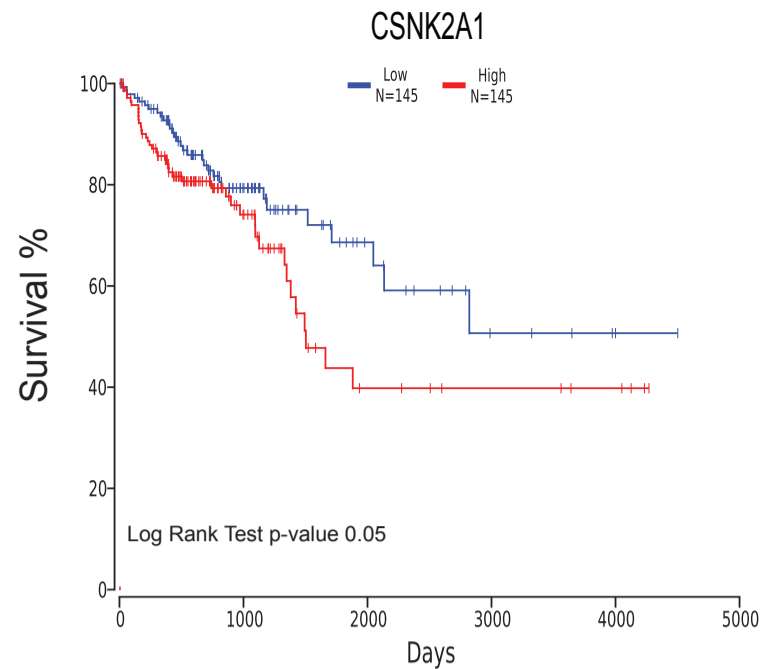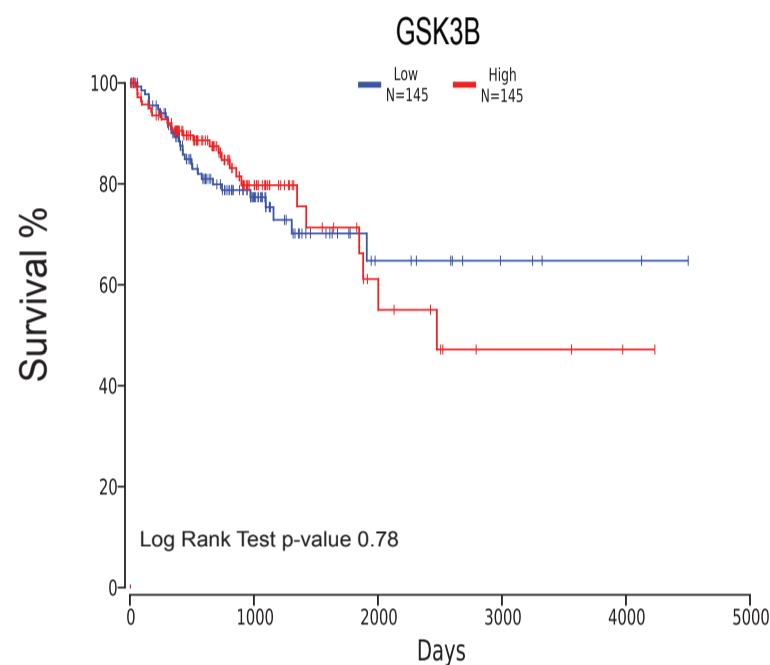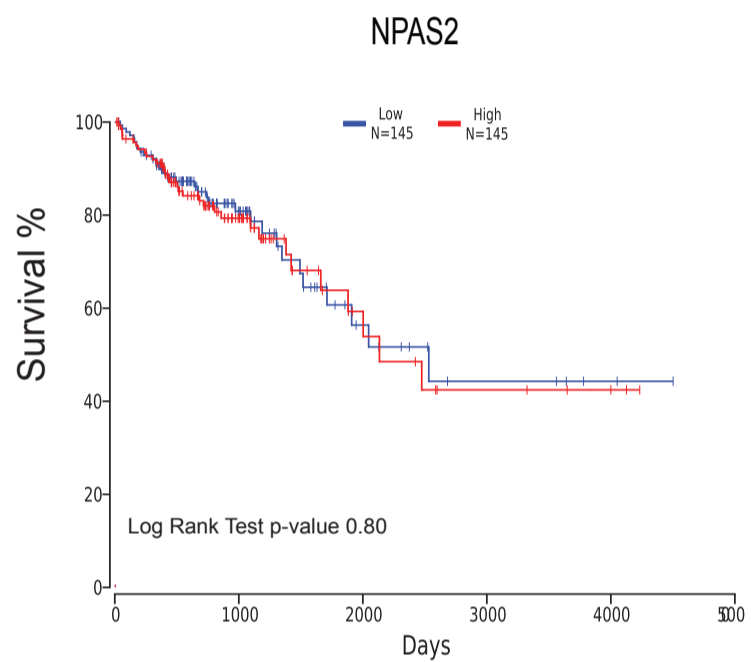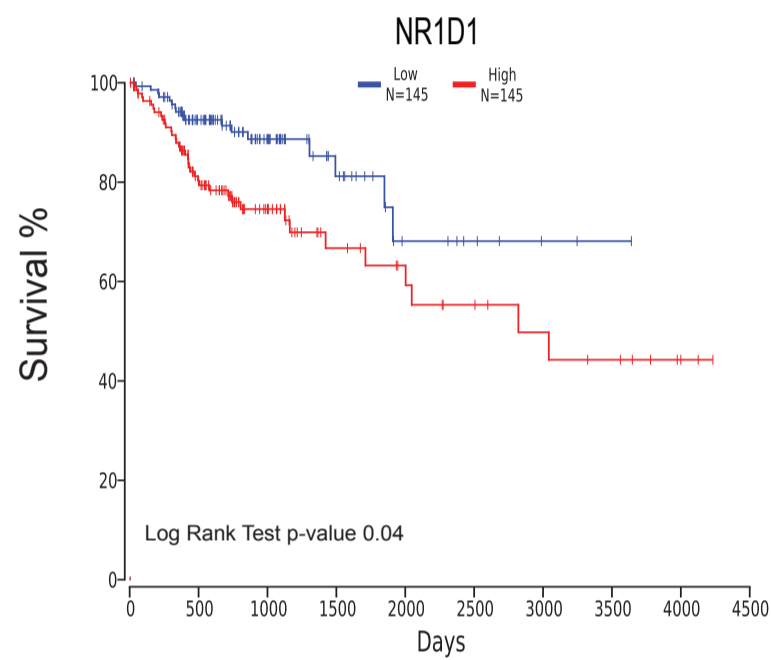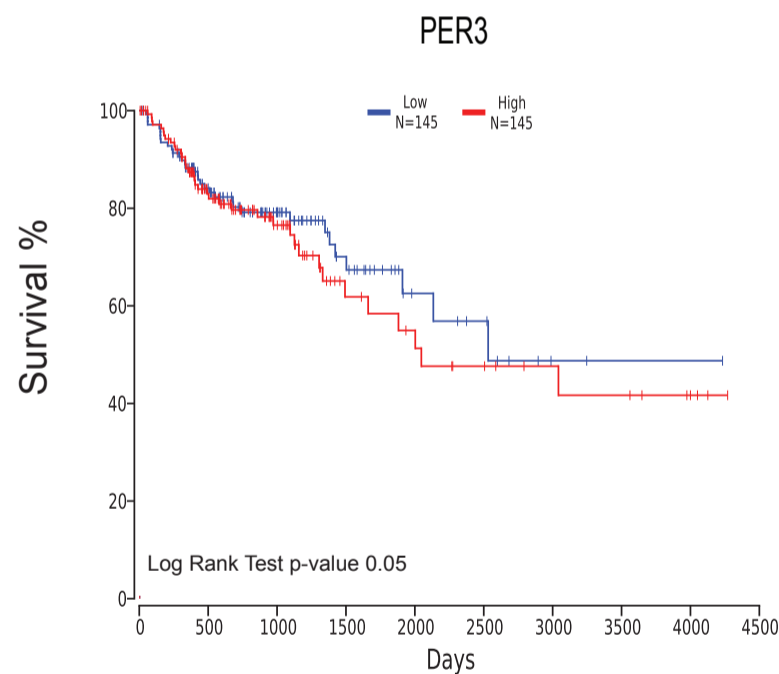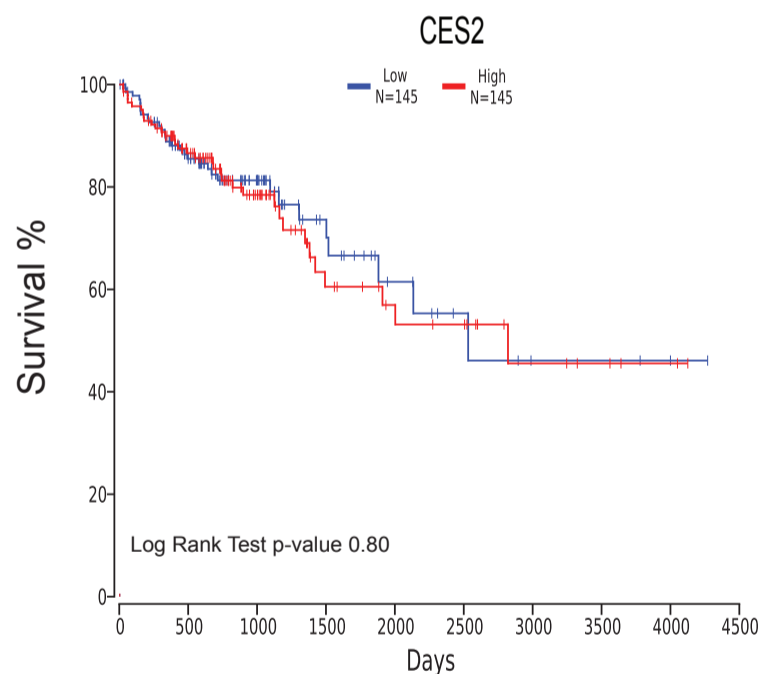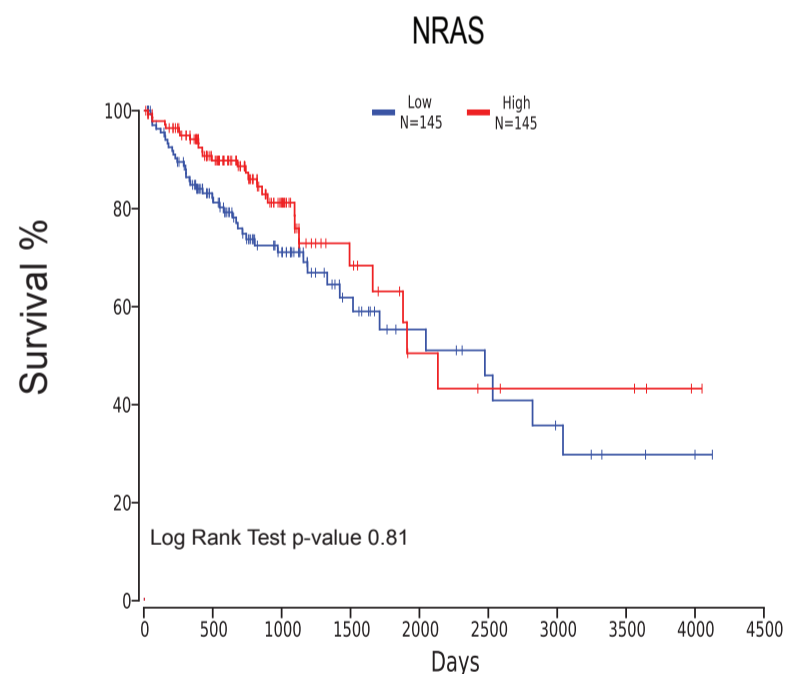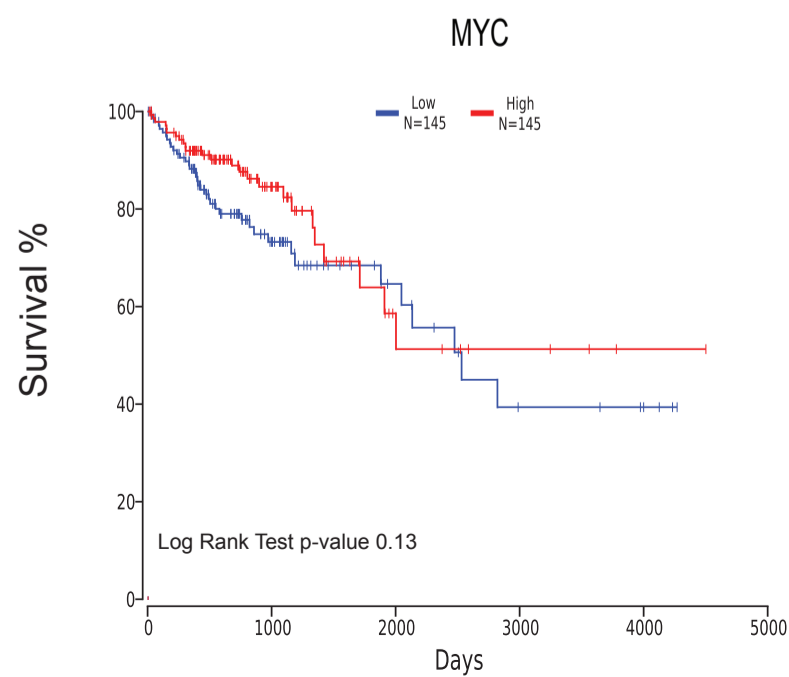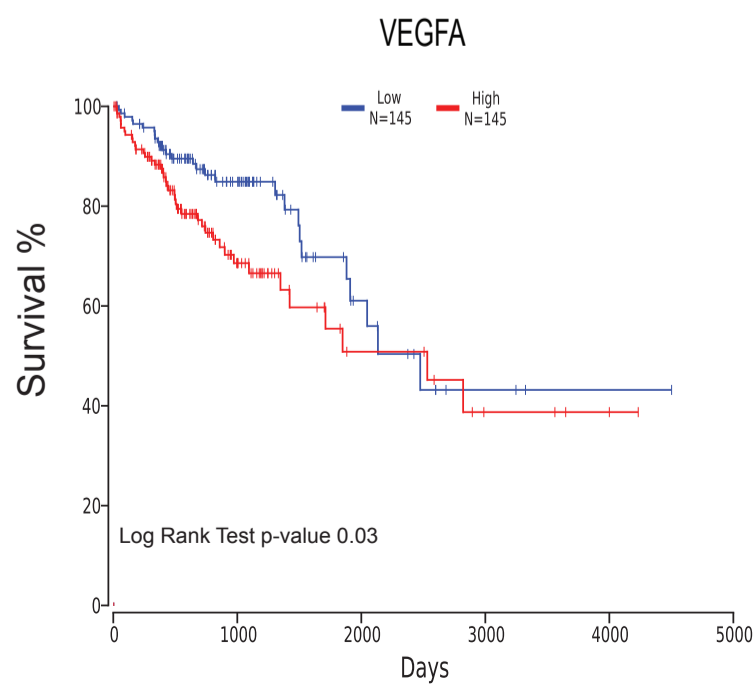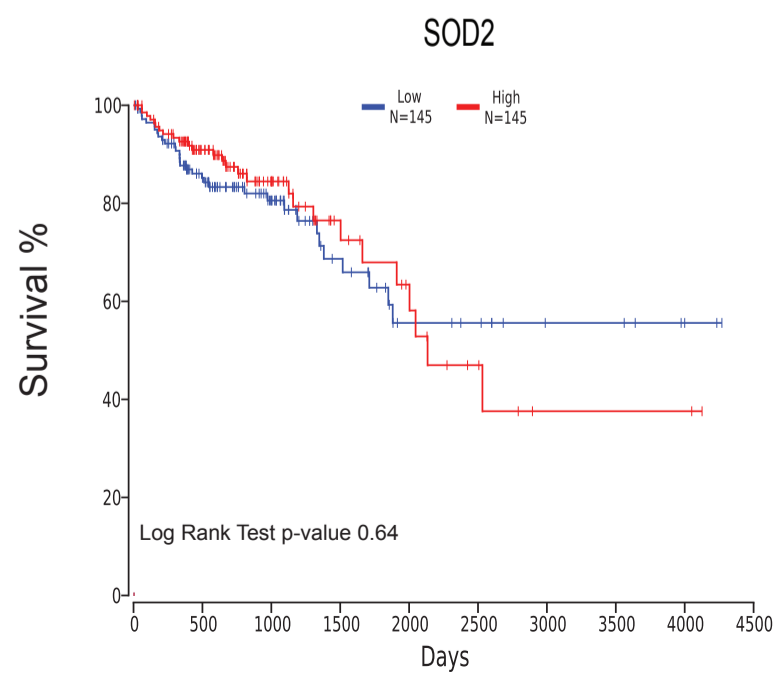

Supplement: Supplementary file 1 [file cancers-12-00963-s001.zip › Supplementary_Material/Figure S5.pdf]
